# Deciphering the mechanism of action of VP343, an antileishmanial drug candidate, in *Leishmania infantum*

**DOI:** 10.1016/j.isci.2023.108144

**Published:** 2023-10-05

**Authors:** Sameh Obeid, Eloisa Berbel-Manaia, Valérie Nicolas, Indira Dennemont, Julien Barbier, Jean-Christophe Cintrat, Daniel Gillet, Philippe M. Loiseau, Sébastien Pomel

**Affiliations:** 1 Université Paris-Saclay, CNRS BioCIS, 91400 Orsay, France; 2 Université Paris-Saclay, UMS-IPSIT, Microscopy Facility, 92019 Châtenay-Malabry, France; 3 Université Paris-Saclay, CEA, INRAE, Département Médicaments et Technologies pour la Santé (DMTS), SIMoS, 91191 Gif-sur-Yvette, France; 4 Université Paris-Saclay, CEA, INRAE, Département Médicaments et Technologies pour la Santé (DMTS), SCBM, 91191 Gif-sur-Yvette, France

**Keywords:** Parasitology, Drugs, Cell biology

## Abstract

Antileishmanial chemotherapy is currently limited due to severe toxic side effects and drug resistance. Hence, new antileishmanial compounds based on alternative approaches, mainly to avoid the emergence of drug resistance, are needed. The present work aims to decipher the mechanism of action of an antileishmanial drug candidate, named VP343, inhibiting intracellular *Leishmania infantum* survival via the host cell. Cell imaging showed that VP343 interferes with the fusion of parasitophorous vacuoles and host cell late endosomes and lysosomes, leading to lysosomal cholesterol accumulation and ROS overproduction within host cells. Proteomic analyses showed that VP343 perturbs host cell vesicular trafficking as well as cholesterol synthesis/transport pathways. Furthermore, a knockdown of two selected targets involved in vesicle-mediated transport, Pik3c3 and Sirt2, resulted in similar antileishmanial activity to VP343 treatment. This work revealed potential host cell pathways and targets altered by VP343 that would be of interest for further development of host-directed antileishmanial drugs.

## Highlights

• Disruption of fusion of *Leishmania* vacuoles with host cell late endosomes/lysosomes
• Interference with membrane fusion regulators
• Perturbation of cholesterol homeostasis

## Graphical abstract

**Figure:**
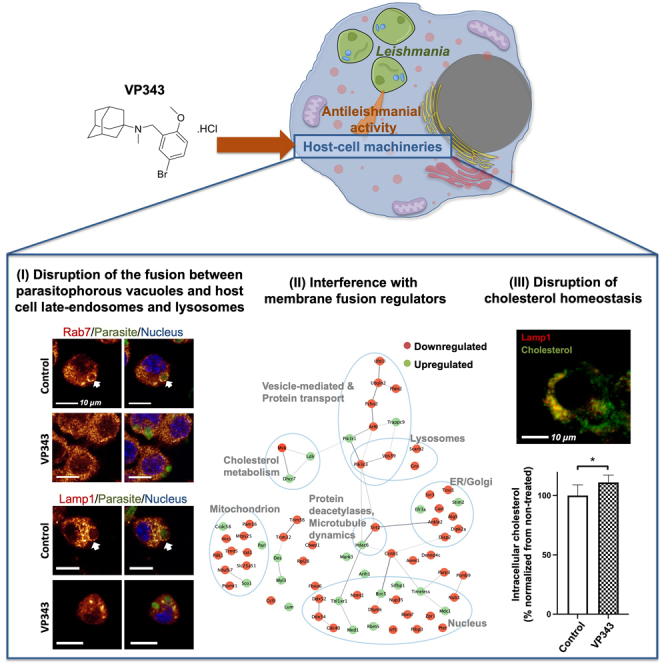

## Introduction

Leishmaniasis is a group of neglected tropical diseases caused by various species of protozoan parasites from the genus *Leishmania* and transmitted by an insect vector, the phlebotomine sandfly. Currently, about 12 million people are affected by the disease worldwide, with approximately 1.3 million new cases and up to 30,000 deaths occurring annually.^1^^,^^2^ Leishmaniases can manifest in three major forms—cutaneous, mucocutaneous, which manifest in localized or diffuse lesions, and visceral leishmaniasis (VL), which affects vital organs such as the liver, spleen, and bone marrow—and can be fatal if left untreated. *Leishmania infantum* is the causative agent of zoonotic VL that affects dogs (Canine Leishmaniasis: CanL) and humans across the Mediterranean Basin, including southern Europe, northern Africa, and parts of Asia. Domestic dogs are the main reservoirs for human infection.^3^ Leishmaniasis is endemic in southern Europe, with an estimated 2.5 million seropositive dogs and ≈700 autochthonous human cases reported each year.^4^^,^^5^

There is no vaccine for humans, and the available studies on the few licensed vaccines for CanL show insufficient efficacy.^6^^,^^7^ Conventional chemotherapy used as first-line treatment for CanL consists of a combined use of pentavalent antimonials or miltefosine with allopurinol.^6^ Treatment of human leishmaniasis is problematic because of the limited arsenal of antileishmanial drugs currently available and is composed essentially in four drugs: pentavalent antimonials, miltefosine, amphotericin B, and paromomycin, the latter being mainly used for cutaneous forms of leishmaniasis.^8^ The use of these drugs has several limitations mainly due to severe toxic side effects, drug resistance, and high cost. Hence, there is a cruel need for the development of new antileishmanial compounds based on alternative approaches mainly to avoid the emergence of drug-resistant parasites. Among these approaches, the development of compounds acting on intracellular parasites, via host cell machineries, seems promising.^9^

During its life cycle, *Leishmania* sp. alternates between an extracellular flagellated form in the insect vector, called the promastigote, and an intracellular form developing within mononuclear phagocytes in the mammalian host, called the amastigote. After being inoculated into the mammalian host through the bite of an infected sandfly, the promastigote is rapidly phagocyted by professional phagocytic cells, including neutrophils and macrophages.^2^ Intracellular promastigotes reside in vacuolar compartments called parasitophorous vacuoles (PVs) where they transform into non-motile amastigotes. *Leishmania*-containing PVs interact extensively with host cell compartments of the endocytic and the secretory pathways (i.e., early and late endosomes, lysosomes, and endoplasmic-reticulum) in order to acquire molecules and nutrients that contribute to parasite maturation, survival, and replication within infected cells.^10^^,^^11^ As a result, PVs acquire sequentially several surface components characteristic of each stage of their maturation process, such as the early endosome antigen 1 (EEA-1), the GTPase Rab7, or the lysosomal-associated membrane proteins 1 (Lamp-1), which are markers of early endosomes, late endosomes, and lysosomes, respectively.^2^

The membrane fusion machinery implicated in the fusion between PVs and host cell compartments (e.g., early endosomes, late endosomes, and lysosomes), which is mainly mediated by soluble N-ethylmaleimide sensitive factor attachment protein receptor (SNARE) proteins and their accessory regulators (e.g., Rab GTPases), is manipulated by *Leishmania* parasites, mainly through their two abundant surface molecules GP63 metalloprotease and LPG, in order to create a safe niche for their replication.^12^ It has been previously shown that knocking down ER/Golgi SNAREs, i.e., sec22b, or some of its known cognate partners (D12, syntaxin-18, and syntaxin-5), results in the reduction of PV size and the inhibition of parasite replication with minimal effects on host cell processes.^13^

In the context of developing a host-directed therapy, an adamantane compound termed ABMA (1-adamantyl (5-bromo-2-methoxybenzyl) amine) was identified, using a cell-based high-throughput screening (HTS), to protect cells from intracellular toxins.^14^^,^^15^ ABMA was shown to exhibit a broad-spectrum activity against intracellular toxins and pathogens.^16^^,^^17^^,^^18^ Particularly, ABMA showed a substantial efficacy against different intracellular bacteria, such as *Chlamydia* or *Simkania*, or viruses, such as Ebola, Dengue-4, HSV-2, and influenza virus. As well, ABMA inhibits the development of the protozoan parasite *L. infantum* with an IC_50_ at ∼7 μM on intramacrophage amastigotes, but with a weak selectivity index (SI) at 3.6. Among a library of 142 ABMA analogues, we previously identified a compound termed VP343, which displayed a higher antileishmanial activity than ABMA, with an IC_50_ value at 0.32 μM, specifically on intracellular *L. infantum* amastigotes *in vitro*, using RAW264.7 macrophage cell model.^19^ In this study, VP343 showed a very interesting SI value at 199, much higher than the reference drug miltefosine displaying an SI value of only ∼8. In addition, this compound presented interesting *in vivo* antileishmanial activity, similar to miltefosine, besides encouraging ADME (Absorption, Distribution, Metabolism, and Excretion) data, making this compound a promising drug candidate for the treatment of VL, especially CanL.^19^ However, the signaling pathways and molecular factors involved at the cellular level in the antileishmanial activity of VP343 have not been determined yet.

In the present work, the mechanism of action of VP343 was investigated on intracellular *L. infantum*, using RAW264.7 macrophage model, where a very interesting antileishmanial activity of the compound was previously described^19^ using cell imaging with several markers of host cell vesicular trafficking to study the influence of VP343 on the cross-talk between host cell endolysosomal compartments and *L. infantum*-containing PVs and to determine how the parasites are eliminated within host cells after treatment. The host cell pathways targeted by VP343 were then identified using mass spectrometry (MS) analysis, and potential targets of the compound were further explored using siRNA. The interference of the compound with cholesterol trafficking and ROS production within host cells, as well as its effect on apoptosis, were also investigated.

## Results

### VP343 exerts prolonged antileishmanial activity by acting through the host cell machinery

A previous work by Pomel et al.^19^ has found an IC_50_ value for VP343 on *L. infantum* intracellular amastigotes of 0.32 μM with a high SI of 199, using RAW 264.7 macrophages as host cells. Interestingly, VP343 had a low activity on *L. infantum* axenic amastigotes, with a mean IC_50_ value of 82.31 μM, reflecting a high specificity of VP343 for the intracellular form of the parasite. Here, we first investigated the cytotoxicity of VP343 on RAW 264.7 macrophages after incubation with different concentrations of the compound for 24 h, 48 h, and 72 h. The CC_50_s of VP343 were determined at 165.5 ± 2.9 μM, 81.8 ± 10.4 μM, and 45.6 ± 6.5 μM after 24 h, 48, h and 72 h of incubation, respectively (Table S1). From these results, a VP343 concentration of 10 μM was selected for cell treatments in the present study, with a maximal incubation time of 24 h postinfection. This concentration should accentuate the antileishmanial effect of VP343 while remaining non-cytotoxic, as it is far from the CC_50_ on RAW 264.7 macrophages even after 72 h of treatment (Table S1). Furthermore, we confirmed that 10 μM of VP343 had no effect on the growth of *L. infantum* promastigotes neither on their differentiation into amastigotes in axenic conditions, with about 80% of the parasites differentiated into amastigotes after 24 h of incubation under axenic condition in the differentiation medium (see STAR Methods; Figures S1A and S1B). In addition, no difference was observed on host cell infection when parasites were treated by VP343 prior to infection in comparison to untreated control, showing that the compound did not affect the virulence of the parasites toward their host cells (Figure S1C). However, when parasites were pre-treated by VP343 and the compound was kept during the course of cell infection, a significant decrease of parasites per host cell was observed in comparison to the control. Together, these results indicate that the antiparasitic effect of the compound relies on a host-cell-directed mechanism of action (Figure S1C).

**Figure 1:**
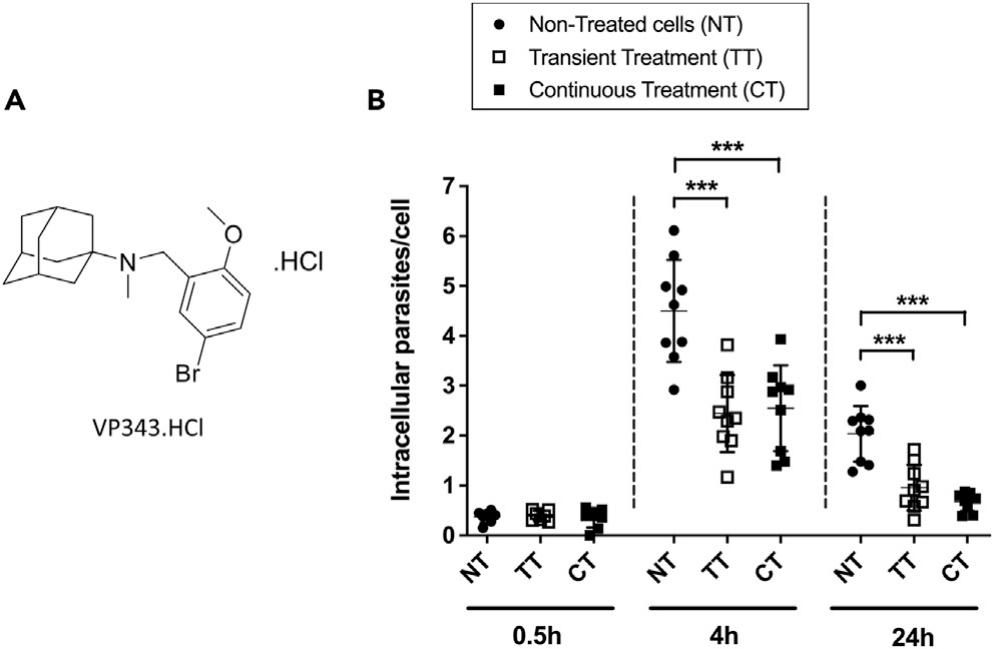
Effect of VP343 on intracellular parasites (A) Chemical formula of VP343. (B) NT (black dots): non-treated cells. TT (transient treatment, open squares) and CT (continuous treatment, closed squares): cells were treated with 10 μM of VP343 for 1 h prior infection. In the TT condition, the compound was removed from cell culture after 1 h incubation before infection. In the CT condition, the compound was maintained during the course of infection. Results represent mean ± SD of three independent experiments, each done in triplicate. Statistical analyses were done using the Student’s t test*.*

The effect of the compound on the survival and proliferation of intracellular *L. infantum* parasites was further evaluated after treatment of RAW 264.7 macrophages with 10 μM of VP343 at 30 min, 4 h, and 24 h postinfection. In control cells, the average number of intramacrophage parasites increased by ∼11 times between 30 min and 4 h postinfection (Figure 1B), from an average of 0.4 parasites/macrophage to 4.5 parasites/macrophage, respectively. The number of parasites within control cells was then reduced after 24 h of infection, to an average of 2 parasites/cell. As the confluence of RAW264.7 macrophages was at 80%–85% at the time of infection in our conditions, the decrease of parasite load between 4 h and 24 h of infection would not be due to an exponential growth of macrophages that would lead to an underestimation of the number of parasites per cell. On the contrary, we postulate that it would be due to a reduced ability of some parasites to survive within macrophages over the first 24 h, in line with the study reported by da Silva Vieira and colleagues^20^ in primary bone-marrow-derived macrophages demonstrating a variable ability among different *Leishmania* species to survive and replicate over time within macrophages. When macrophages were treated with 10 μM VP343 for 1 h prior to their infection with *L. infantum*, a reduction by about 45% and 60% of the parasite load was observed at 4 h and 24 h postinfection, respectively, whether the compound was kept in the culture medium during the course of infection or removed prior to infection (Figure 1B). Together, these results confirm that VP343 exhibits its antileishmanial activity by acting through the host cell machinery. Moreover, the compound seems to exert a prolonged antileishmanial activity as the decrease of parasite load was observed even at 24 h postinfection when VP343 was removed prior to infection. Based on these results, the mechanism of action of VP343 was further investigated in the current study using cells that were incubated with the compound for 1 h prior to their infection in order to ensure an effect on *Leishmania* at the earliest stages of infection, i.e., installation of the parasites within the PVs.

### VP343 has an effect on the interaction of parasitophorous vacuoles with host cell endolysosomal compartments

**Figure 2:**
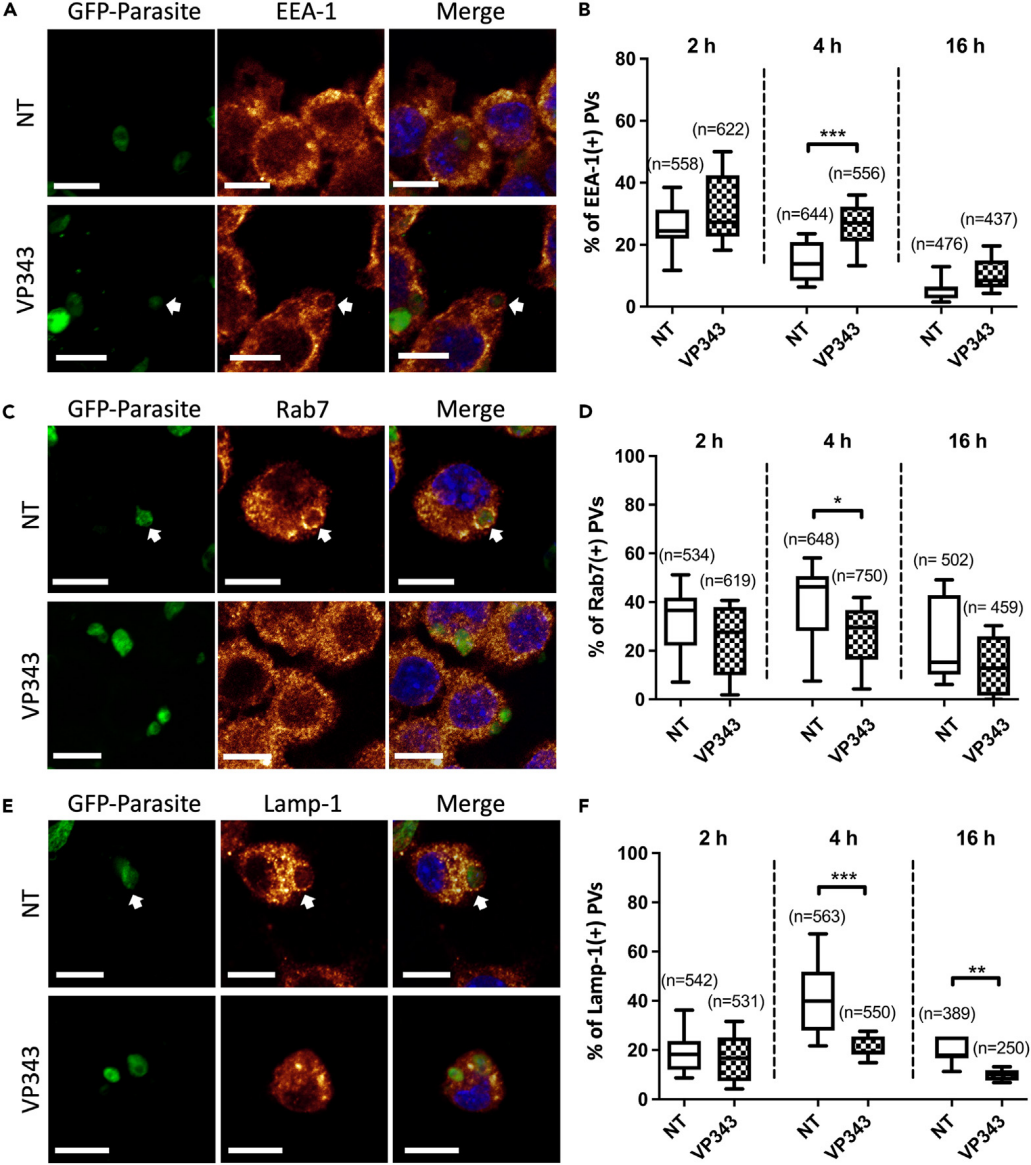
Recruitment of EEA-1, Rab7, and Lamp-1 by PVs harboring *L. infantum* parasites Macrophages, which were either untreated (NT) or treated with 10 μM of VP343, and infected with *L. infantum* amastigotes, were processed for immunofluorescence and examined by confocal microscopy at 2 h, 4 h, and 16 h postinfection. Representative images for EEA-1, Rab7, and Lamp-1 are presented in (A), (C), and (E), respectively. Arrows indicate positive recruitment of the trafficking markers to the PVs. Panels B, D, and F show the percentage of PVs displaying a positive recruitment of EEA-1, Rab7, or Lamp-1, respectively, at 2 h, 4 h, and 16 h postinfection. The results represent two independent experiments, each done in duplicate. “n” represents the number of analyzed PVs. Scale bar: 10 μm. Statistical analyses were done using the Student’s t test.

To examine the effect of VP343 on the maturation of *L. infantum*-containing PVs, we assayed the level of association of PVs with specific markers of early endosomes (i.e., EEA-1), late endosomes (i.e., Rab7), and lysosomes (i.e., Lamp-1), at 2 h, 4 h, and 16 h postinfection. Confocal microscopy imaging showed a significantly higher number of PVs displaying EEA-1 in VP343-treated cells (i.e., 26%) compared with non-treated cells (i.e., 14%) at 4 h postinfection (Figures 2A and 2B). A similar tendency was observed at 16 h postinfection but was not statistically significant. *L. infantum*-containing PVs showed a significantly lower recruitment of the late endosomal marker Rab7 at 4 h postinfection in VP343-treated cells (i.e., 27%) in comparison to the control (i.e., 40%), with also a tendency of decrease after 16 h of infection (Figures 2C and 2D). Similarly, the number of PVs displaying the lysosomal marker Lamp-1 was significantly lower in VP343-treated cells compared with control cells at 4 h (i.e., 21% and 41%, respectively) and 16 h (i.e., 10% and 19%, respectively) postinfection (Figures 2E and 2F). These results suggest that VP343 restricts the interaction of PVs to early endosomes during the first hours of infection and delays the recruitment of late endosomal and lysosomal markers, which seems to reflect an impaired fusion of PVs with late endosomes/lysosomes. However, no effect of the VP343 treatment on the expression level of the host cell endolysosomal markers, i.e., EEA-1, Rab7, and Lamp-1, was observed (Figure S2).

### VP343 interferes with host cell intracellular cholesterol transport but not with its trafficking to the PV

**Figure 3:**
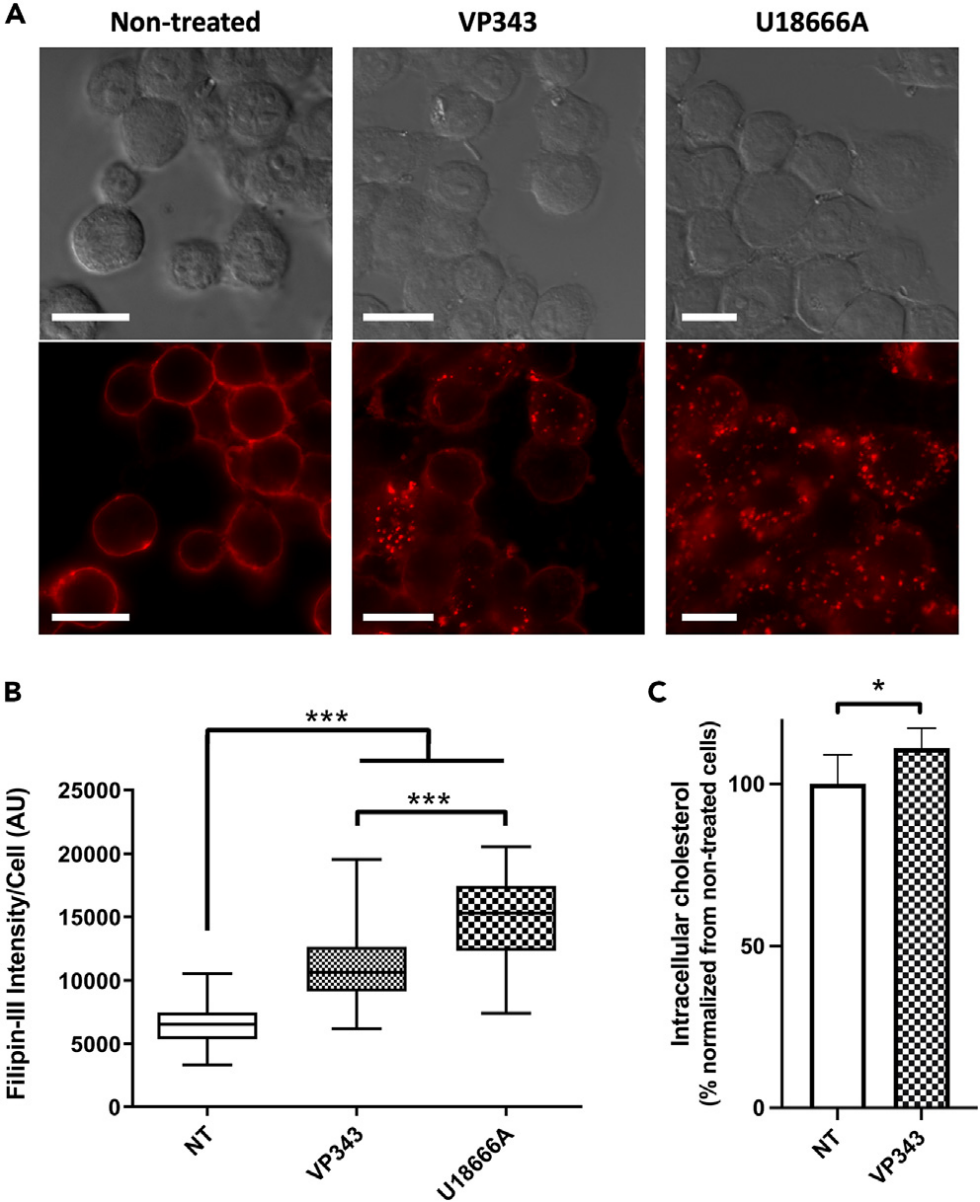
VP343 induces cholesterol accumulation in RAW264.7 (A) RAW 264.7 macrophages untreated (NT) or treated for 5 h with 10 μM of VP343 or U186661 were fixed and incubated with Filipin III for cholesterol labeling. Scale bar: 20 μm. (B) The relative cytoplasmic fluorescence intensity of Filipin III measured by confocal imaging in the untreated control (NT) and in RAW 264.7 macrophages treated with VP343 and U18666A; n = 53 cells for each condition. (C) The intracellular cholesterol content in control (NT) and VP343-treated (VP343) cells. Values represent mean ± SD of triplicate measurements. Statistical analyses were done using the non-parametric Mann-Whitney’s test for (B) and Student’s t test for (C).

**Figure 4:**
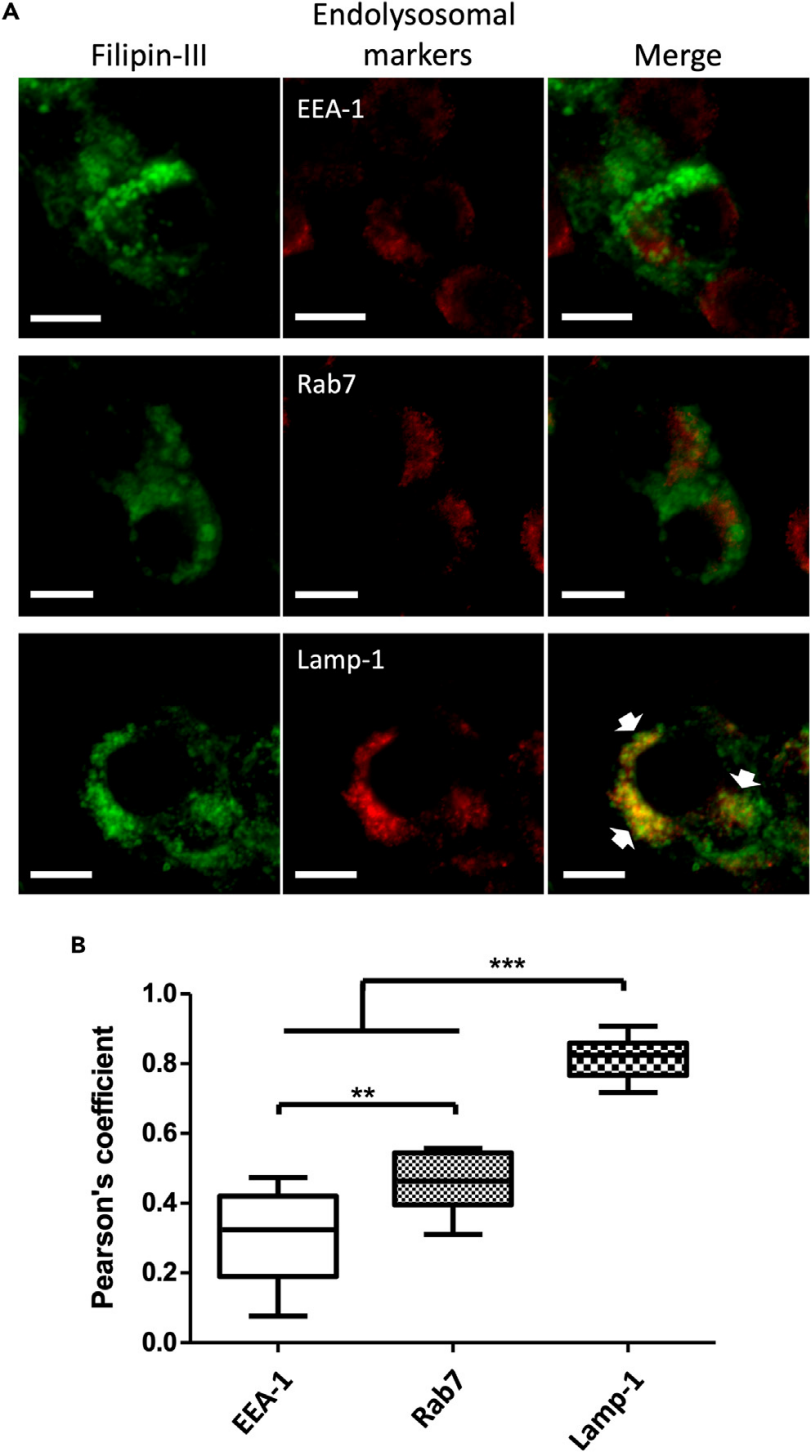
Cholesterol accumulated mainly in Lamp-1-positive compartments within VP343-treated cells (A) Confocal images showing macrophages treated with 10 μM of VP343, fixed, incubated with Filipin III, and further immunolabeled with anti-EEA-1, anti-Rab7, or anti-Lamp-1. White arrows indicate colocalization of free cholesterol with Lamp-1. Scale bar: 10 μm. (B) Pearson’s correlation coefficient of the colocalization of free cholesterol with EEA-1, Rab7, or Lamp-1; n = 30 cells for each condition. Statistical analyses were done using the Student’s t test.

Because the trafficking of intracellular cholesterol passes through the endolysosomal compartments prior to its transport to other subcellular sites,^21^ we investigated whether the intracellular cholesterol transport is impaired in VP343-treated cells. Untreated and VP343-treated cells were incubated with Filipin III, a fluorescent probe that labels free cholesterol (i.e., non-esterified cholesterol). Cells that were treated with U18666A, an inhibitor of intracellular cholesterol transport, were used as positive control. In non-treated cells, Filipin III labeling was mainly located at the plasma membrane (Figure 3A). In cells that were treated with VP343 for 5 h, corresponding to the period when the first alterations of endolysosomal marker recruitment were observed (Figure 2), an accumulation of cholesterol was observed in the cytoplasm and resulted in a significant increase in the fluorescent signal compared with non-treated cells (Figures 3A and 3B). Nonetheless, cholesterol accumulation was more pronounced when cells were treated with the positive control U18666A (Figures 3A and 3B). Likewise, the quantification of intracellular cholesterol also showed a slight but significant increase (i.e., ∼10%) in the total cholesterol content in VP343-treated cells compared with non-treated cells (Figure 3C). Furthermore, when cells were double labeled with Filipin III and markers of the endolysosomal compartment, i.e., EEA-1, Rab7, or Lamp-1, a quantitative analysis showed a high degree of colocalization of Filipin III with Lamp-1, as well as, but to a lower extent, with Rab7, with average Pearson’s correlation coefficients of 0.8 and 0.5, respectively (Figure 4). Moreover, a low colocalization was noticed with EEA-1, with an average Pearson’s coefficient of 0.3. Together, these results show that VP343 treatment interferes with intracellular cholesterol transport and induces its accumulation within lysosomes.

Moreover, we checked whether VP343 interferes with the trafficking of cholesterol to PV containing *L. infantum* by analyzing the accumulation of free cholesterol around the intracellular parasites at 5 h post-infection using Filipin III. The accumulation of free cholesterol around intracellular parasites, detected as a Filipin halo around the PV, was previously described to begin very early (i.e., 1 h postinfection) after the infection and to occur with species that live in communal and individual PVs, such as *L. amazonensis* and *L. infantum*, respectively.^22^ A quantitative analysis of PVs stained with Filipin showed no difference in cholesterol accumulation around parasites between VP343-treated and non-treated cells (Figure S3), suggesting that cholesterol transport to PVs is unaltered in VP343-treated cells.

### VP343 affects the expression of several host cell proteins potentially involved in *L. infantum* infection

**Figure 5:**
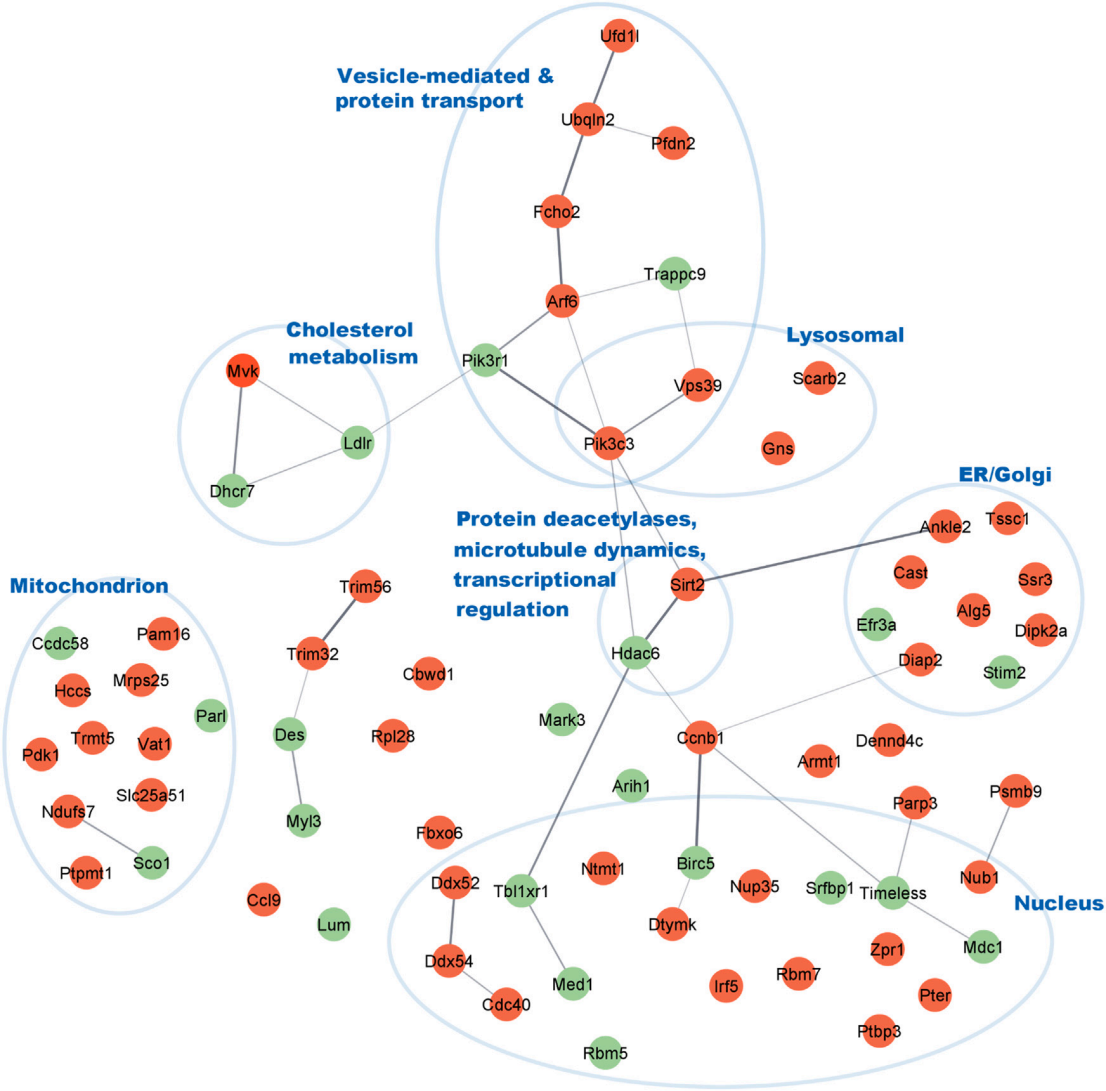
Comparative proteomic analysis of untreated and VP343-treated macrophage extracts Extracts of RAW 264.7 macrophages prepared from untreated and VP343-treated cells (at 5 h posttreatment) were analyzed using MS analysis. The analysis was done in quadruplicate for each condition. The protein network shows the 72 differentially expressed proteins between untreated and VP343-treated macrophages. Green and red circles represent upregulated and downregulated proteins in VP343-treated macrophages compared with untreated cells, respectively. The proteins were distributed depending on their biological process/subcellular location in the cell. The thickness and opacity of the lines linking interacting nodes are proportional to the combined confidence score for the protein association based on the STRING database.

Quantitative proteomic MS was used to profile the proteome changes in VP343-treated cells and to identify the host cell pathways targeted by the compound. Because VP343 activity passes through the host cell machinery, and in order to avoid any potential interference between host cell and parasite proteomes, the proteomic analysis was performed on non-infected macrophages treated or not with VP343 for 5 h or 16 h. Among the 4,450 identified proteins, statistical analysis showed that about 1.1%, i.e., 50 proteins, were significantly downregulated (Table S2**)**, whereas only 0.5%, i.e., 22 proteins, were significantly upregulated (Table S3**)** in cells at 5 h posttreatment in comparison to non-treated cells (Figure 5). At 16 h posttreatment, the level of downregulated proteins dropped to about 0.4% (Table S4), whereas the level of upregulated proteins remained stable (∼0.5% of the total proteins) compared with 5 h treatment (Table S5**)**. A particular attention was devoted to the targeted proteins at 5 h posttreatment as all our analyses showed an effect of VP343 in the first hours after host cell treatment.

Among the 72 misregulated proteins at 5 h posttreatment, we identified a cluster of 24 connected proteins associated with different pathways including vesicular trafficking and the regulation of cholesterol metabolism (Figure 5). Several proteins involved in membrane fusion and protein trafficking between endosomal and lysosomal compartments were significantly downregulated. This was the case for Scarb2, Pik3c3/Vps34, Fcho2, DipK2a, Ubqln2, Arf6, and Vps39/Vam6 (Figure 5).^23^^,^^24^^,^^25^^,^^26^^,^^27^^,^^28^^,^^29^^,^^30^ These results indicate a defect in the endolysosomal trafficking machinery in VP343-treated cells. In addition, significant changes were observed at 5 h posttreatment in the level of proteins involved in cholesterol synthesis, i.e., MvK, Dhcr7,^31^ and transport, i.e., Scarb2.^32^ Moreover, the protein deacetylase Sirt2, which is part of the cluster of the 24 differentially expressed and interconnected protein network (Figure 5), was among the most downregulated proteins (i.e., ≈ 6-fold) (Table S2). Furthermore, at 5 h and 16 h posttreatment of macrophages, the low-density lipoprotein receptor (LDL-R) was consistently upregulated compared with non-treated cells (Tables S3 and S5).

### Knockdown of Mvk, Scarb2, Pik3c3, or Sirt2 inhibits intracellular *L. infantum* survival and/or decreases Lamp-1 recruitment to PVs

**Figure 6:**
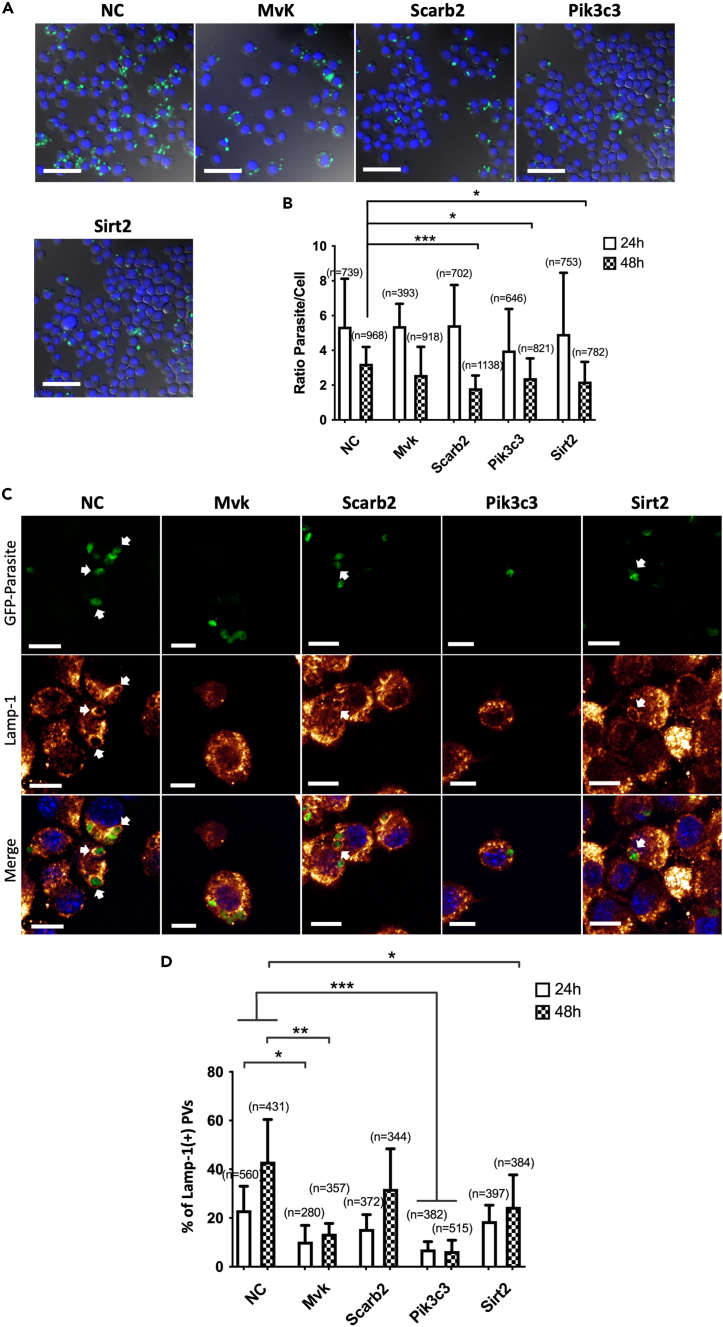
Role of Scarb2, MvK, Pik3c3, and Sirt2 in the intracellular survival of *L. infantum* and recruitment of Lamp-1 to PVs (A) Representative microscopy images showing macrophages that were treated with control siRNA (NC) or siRNA targeting Mvk, Scarb2, Pik3c3, or Sirt2, at 48 h postinfection with *L. infantum* (green). Cell nuclei (blue). Scale bar: 50 μm. (B) Parasite load in macrophages at 24 h and 48 h postinfection after treatment by the different siRNA. “n” represents the number of analyzed cells. (C) Representative confocal microscopy images showing the recruitment of Lamp-1 to PVs in cells treated by the different siRNAs, at 48 h postinfection with *L. infantum*. Representative PVs associated with Lamp-1 recruitment are indicated by white arrows. (D) Proportion of PVs positive for Lamp-1 after siRNA treatment at 24 h and 48 h postinfection. “n” represents the number of analyzed PVs. The results in (B) and (D) represent means ± SD of two independent experiments, each done in duplicate. Scale bar: 10 μm.

Four potential VP343 targets (i.e., Mvk, Scarb2, Pik3c3, or Sirt2), identified by proteomic analysis, were knocked down by transfection of macrophages with specific siRNA prior to their infection with *L. infantum* in order to determine their role in parasite survival, Lamp-1 recruitment, and thus in the mechanism of action of VP343. These selected potential targets were identified by MS analysis to be downregulated by more than 2.5-fold in VP343-treated cells in comparison to the control (Table S2) and were chosen from different host cell pathways based on their association with late endosomal/lysosomal compartments (i.e., Scarb2, Pik3c3), with microtubule remodeling (i.e., Sirt2) and cholesterol biosynthesis and transport (i.e., Mvk, Scarb2).^23^^,^^25^^,^^31^^,^^32^^,^^33^ No significant decrease of parasite load was observed at 24 h post-infection in siRNA-treated macrophages in comparison to the control (Figures 6A and 6B). Interestingly, at 48 h post-infection, a significant decrease of parasite load of 44%, 32%, and 26% was observed after knockdown of Scarb2, Pik3c3, and Sirt2, respectively (Figures 6A and 6B). However, no change in the ratio of parasites per host cell was noticed after Mvk knockdown (Figures 6A and 6B).

As the recruitment of Lamp-1 to the PV was impaired following VP343 treatment at 4 h and 16 h postinfection (Figure 2F), the localization of this lysosomal marker was also analyzed in knockdown macrophages (Figures 6C and 6D). The results show that the knockdown of Mvk and Pik3c3 resulted in a significant decrease in the number of PVs displaying Lamp-1 at both 24 h and 48 h postinfection in comparison to the control cells (Figures 6C and 6D). When Sirt2 was knocked down, a significant decrease in the recruitment of Lamp-1 was also observed at 48 h postinfection (Figures 6C and 6D). However, no changes in the proportion of Lamp-1-positive PVs were observed after knockdown of Scarb2 in comparison to control cells (Figures 6C and 6D).

### VP343 induces an increase of host cell ROS production but does not trigger early apoptosis-like cell death in intracellular parasites

**Figure 7:**
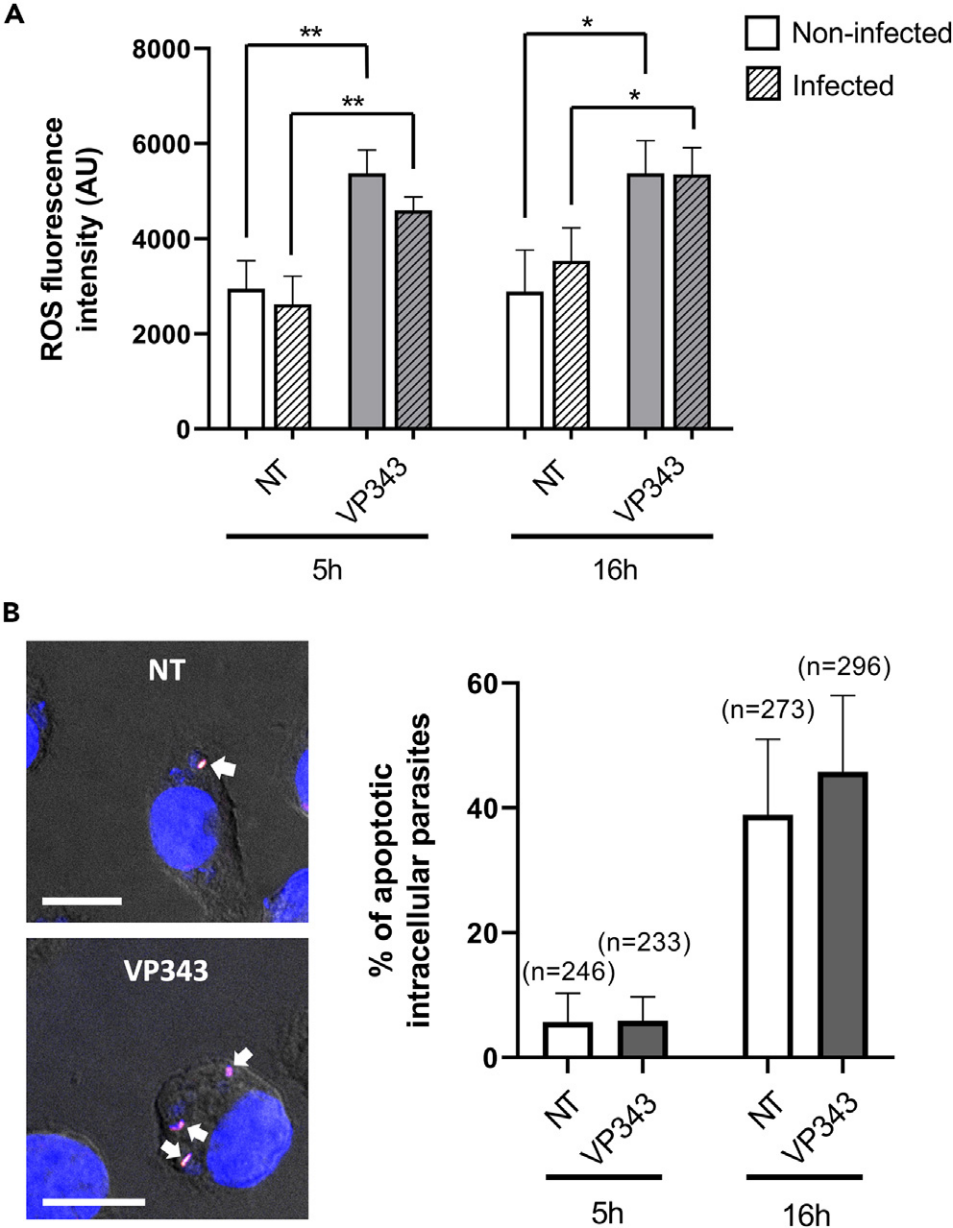
ROS production in VP343-treated cells and intracellular *Leishmania* apoptosis (A) *L. infantum* infected or uninfected live macrophages treated or not (NT) with 10 μM VP343 were stained with CellRox Deep Red reagent to quantify intracellular ROS production. The measurement was done at 5 h and 16 h posttreatment of infected and uninfected cells. Data represent means ± SD of triplicate measurements. (B) Intracellular *L. infantum* apoptosis was assessed at 5 h and 16 h postinfection in VP343-treated and untreated cells (NT) using TUNEL staining to detect DNA breaks in the parasites. The white arrows in the representative images of the left panels indicate intracellular parasites labeled with TUNEL. The right panel shows the proportion of intracellular apoptotic parasites in each condition. Data represent means ± SD of triplicate measurements; “n” represents the number of analyzed parasites. Scale bar: 10 μm. Statistical analysis was done using the Student’s t test.

The intracellular ROS level was quantified in macrophages either uninfected or infected with *L. infantum* in the presence or absence of VP343. The results showed that VP343 treatment induced a significant increase by ≈ 40% of intracellular ROS production at 5 h and 16 h posttreatment in both infected and non-infected cells (Figure 7A). The level of ROS in VP343-treated cells was stable at 5 h and 16 h posttreatment whether macrophages were infected or not.

Moreover, the induction of apoptosis-like cell death was explored in intracellular *L. infantum* within VP343-treated cells by assessing DNA fragmentation using TUNEL assay. The results showed no significant difference in the level of DNA fragmentation in intracellular parasites between untreated and VP343-treated cells at both 5 h and 16 h postinfection (Figure 7B). However, DNA fragmentation in intracellular parasites increased significantly at 16 h postinfection (i.e., ∼ 40%), independently of the treatment, in comparison to 5 h postinfection (i.e., ∼ 6%), in agreement with the data presented in Figure 1B showing less intracellular parasites at 24 h postinfection than at 4 h postinfection.

## Discussion

The novel approach for the treatment of leishmaniasis consisting in finding compounds that interfere with host cell machineries involved in *Leishmania* infection seems to be promising to prevent emergence of drug resistance. In a previous study realized in our research team,^19^ an adamantane derivative, termed VP343, was identified to exhibit interesting *in vitro* and *in vivo* antileishmanial activities on *L. infantum* with interesting ADME data making this compound a drug candidate for the treatment of visceral leishmaniasis. The mechanism of action of VP343 was investigated at the cellular level in the present work.

Firstly, no effect of the compound was observed on the virulence of axenic *L. infantum* forms, in agreement with our previous data reporting a low activity of the compound on axenic amastigotes (i.e., IC_50_ ≈ 82.31 μM).^19^ Moreover, the CC_50_ determined in this study at 48 h (i.e., 81.8 ± 10.4 μM) was in agreement with the value of 63.7 ± 4.4 μM previously reported.^19^ Our results showed that treatment of macrophages with VP343 for 1 h before their infection with *L. infantum* was as efficient as when the compound was continuously present during the course of infection. In addition, VP343 treatment exerted a long-lasting antileishmanial activity, at least for 24 h, even in the absence of the molecule during the course of infection. These results show that the mechanism of action of VP343 operates through the host cell machinery.

Furthermore, our data showed that VP343 hampered the recruitment of Rab7 and Lamp-1 to *L. infantum*-containing PVs from 4 h postinfection. These results indicate that VP343 would induce a defect in the interaction between PVs and late endosomes/lysosomes that may affect the maturation course of the PVs, leading to the elimination of *Leishmania* by host cells.^11^

Interaction of PVs, in particular amastigotes-containing vacuoles, with late endosomes and lysosomes has been shown to occur early after the parasite entry into host cells.^11^^,^^34^^,^^35^^,^^36^^,^^37^ During the first hour postinfection, the majority of amastigote-containing PVs (>80%) have been reported to include soluble and membrane molecules associated with late endosomes/lysosomes, such as Rab7, macrosialin, Lamp-1, and cathepsin proteases.^34^^,^^37^ Hence, the interaction of PVs with late endosomes/lysosomes seems to be crucial to create a favorable niche for the survival of intracellular *Leishmania*. The disruption of this interaction by VP343 could be detrimental for parasite installation within its vacuole.

Furthermore, the MS analysis showed a higher number of differentially expressed proteins at 5 h posttreatment (i.e., 72 proteins) compared with 16 h posttreatment (i.e., 40 proteins), reflecting a higher activity of VP343 on the host cell during the first hours of infection, when the parasite settles within its vacuole. This result is in accordance with the results showing at 5 h posttreatment an antileishmanial activity (Figure 1B), a default in the recruitment of late endosomal and lysosomal markers (Figure 2), cholesterol accumulation (Figures 3 and 4), and ROS production (Figure 7A). Hence, particular attention was devoted in the current work to the proteins differentially expressed at 5 h posttreatment with the aim to identify the host-cell molecular target(s) of VP343 (Figure 5).

The expression of several proteins associated with cholesterol biosynthesis and transport (i.e., LDL-R, Mvk, Dhcr7, and Scarb2) was altered by VP343 treatment. In particular, LDL-R was consistently upregulated 5 h and 16 h posttreatment of macrophages, compared with non-treated cells. The transporter LDL-R is involved in the uptake of low-density lipoprotein (LDL) to maintain the cholesterol homeostasis in cells.^38^ Interestingly, Manzano et al.^39^ showed that *L. infantum* modulates cholesterol pathways in host macrophages, including a downregulation of LDL-R and other lipoprotein receptors, resulting in cholesterol content modification in plasma membrane that may contribute to immune evasion and survival of the parasites. Hence, a higher expression of LDL-R, as it was observed in the case of VP343-treated cells, may play a role in shaping the host immune response to eliminate the parasite, in addition to its role in cholesterol homeostasis.

On the other hand, VP343 was shown to induce accumulation of intracellular cholesterol in host cell lysosomal compartments displaying Lamp-1 associated with an increase in the total cholesterol content in comparison to non-treated cells. Together, these results suggest that VP343 disrupts the cholesterol homeostasis in host macrophages. However, we observed no difference in the accumulation of cholesterol in *L. infantum*-containing PVs whether cells were treated or not by VP343 (Figure S3). Thus, it is unlikely that the antileishmanial activity of VP343 would be related to a defect in the recruitment of cholesterol to the PVs. Of note, intracellular *Leishmania* can take up cholesterol from the host-cell membrane^22^ or by direct fusion with endocytic vacuoles containing LDL particles.^40^ Otherwise, cholesterol and sphingolipids are important regulators of lysosomal membrane trafficking and fusion. The SNARE complexes, which mediate membrane fusion, are largely associated with cholesterol-rich domains in membranes.^41^^,^^42^ The endolysosomal accumulation of cholesterol has been reported to result in lysosomal SNARE sequestration in cholesterol-enriched membranes and to interfere with the late endosome/lysosome fusion.^43^

It is worth noting that the induction of intracellular cholesterol accumulation by VP343 is comparable to that shown in cells treated with ABMA.^15^ However, in the case of ABMA, it was suggested that this accumulation occurred in late endosomes, whereas here the cholesterol accumulated mainly in lysosomes. Furthermore, ABMA has been shown to induce accumulation of Rab7-positive late endosomal compartments^15^ and delay endolysosomal trafficking of endocytosed cargoes, by trapping them in Rab7-positive compartments,^17^ suggesting a distinct mode of action of VP343 on the host cell intracellular trafficking pathways.

Among the host-cell proteins found by the MS analysis to be significantly downregulated upon VP343 treatment, four proteins were selected for analysis by siRNA (i.e., Sirt2, Mvk, Scarb2, and Pik3c3). These proteins were selected based on their implication in vesicle-mediated transport within the endolysosomal compartments (i.e., Scarb2, Pik3c3, Sirt2) or in cholesterol biosynthesis and transport (i.e., Mvk, Scarb2).^23^^,^^25^^,^^31^^,^^32^^,^^33^ The results showed that knockdown of Scarb2, Pik3c3, and Sirt2 resulted in a significant decrease of the parasite load in comparison to control cells after 48 h of infection. Furthermore, the antileishmanial activity of Pik3c3 and Sirt2 was associated with a significant decrease in the number of PVs displaying Lamp-1, similar to VP343.

A study by Jaber and colleagues^25^ reported that deletion of Pik3c3 (Vps34), the class III phosphoinositide 3-kinase (PI3K), disrupts late endosomal trafficking and blocks lysosomal maturation due to defective recruitment of the Rab7 GTPase-activating protein (GAP) Armus to late endosomes.^25^ On the other hand, Sirt2, which is a member of the sirtuin family of nicotinamide-adenine-dinucleotide (NAD)-dependent deacetylases, is predominantly found associated with microtubules not only in the cytoplasm but also in the nucleus. Sirt2 can selectively deacetylate α-tubulin and histone H3 (H3K18), resulting in controlling microtubule dynamics and epigenetic regulation of genes within host cells, respectively.^33^^,^^44^^,^^45^ Microtubule network has been shown to provide tracks for the movement of endolysosomal compartments, mediated by dynein-dependent mechanism, followed by their fusion regulated by SNAREs.^46^^,^^47^ Moreover, Sirt2 deficiency has been shown to cause an impaired bacterial infection,^45^^,^^48^ suggesting to be the result of epigenetic regulation of a subset of host cell genes that are necessary for bacterial infection. These roles in regulating late endocytic trafficking reported for Pik3c3 and Sirt2 are in agreement with our results showing that the antileishmanial activity resulted from the knockdown of one of these targets is associated with a defect in the fusion between lysosomes and PVs.

Moreover, Scarb2 (also known as Limp-2) is a glycoprotein located in the membranes of lysosomes and late endosomes. It is believed to exert various functions^23^ such as the biogenesis and maintenance of late endosomes and lysosomes^49^ and to participate in lysosomal cholesterol egress.^21^^,^^32^ Scarb2 depletion was shown to promote a lysosomal cholesterol accumulation associated with an upregulation of LDL-R expression,^32^ in agreement with our data. However, our results showed that the antileishmanial activity obtained from the knockdown of Scarb2 was not associated with a significant hindrance of Lamp-1 recruitment to PVs despite the lower proportion of PVs positive for Lamp-1 at 24 h and 48 h postinfection compared with control cells. This may suggest that the antileishmanial activity resulting from Scarb2 targeting is not directly mediated by the blockage of the cross-talk between lysosomes and PVs as observed after VP343 treatment. Furthermore, Mvk knockdown resulted in a decrease in Lamp-1 recruitment to PVs but without an effect on the parasite load. This disruption of Lamp-1 recruitment to PVs may be the result of the interference with cholesterol biosynthesis upon Mvk knockdown, as previously described.^31^ However, the absence of antileishmanial activity after knockdown of Mvk indicates that this enzyme may not be directly targeted by VP343 during its mechanism of action and that targeting of lysosomal fusion with PVs alone may not be sufficient for an efficient elimination of *L. infantum*. Together, our siRNA analysis may suggest that the anti-leishmanial effect of VP343 is the result of a multitarget action.

Furthermore, our results showed that VP343 induces ROS production within host cells independently of their infection status. ROS generation is a major strategy used naturally by macrophages to eliminate intracellular pathogens.^11^ Many compounds (e.g., antimonials) have been described to exert antileishmanial activity through the induction of ROS in host cells.^50^^,^^51^^,^^52^^,^^53^ It is interesting to note that the induction of ROS by VP343 is apparently non-toxic to host cells because the compound, at the concentration of 10 μM, is far from being toxic. However, supplementary analyses of ROS production should be considered, especially in a dose-dependent manner of VP343, before further development of the molecule for the treatment of leishmaniasis.

The analysis of DNA fragmentation in intracellular *L. infantum* showed that TUNEL labeling is mainly observed in the kinetoplasts, suggesting breakage of the kinetoplast DNA prior to nuclear DNA in apoptotic parasites, in agreement to the report by Das and collaborators*.*^54^ Our results showed similar levels of DNA fragmentation in intracellular *L. infantum* whether cells were treated or not by VP343, suggesting that the compound may trigger cell death pathways distinct from apoptosis in parasites (e.g., necrosis).^55^ The triggered cell death mechanisms depend not only on the type of stimuli but also on the treatment dose and the exposure time.^56^ Also, the mechanistic boundaries between the different types of cell death are currently not well determined in *Leishmania* sp., and cross-talks between the pathways exist.^56^ Finally, despite the advances in our understanding of *Leishmania* cell death,^55^ such an *in situ* evaluation on intracellular parasites is currently challenging at the technical level.

Altogether, our results suggest that VP343 acts probably on several host cell targets to exert its antileishmanial activity. This study shed light on the mechanistic action of VP343 and identified potential targets of the compound having several roles within the host cell, particularly in vesicle-mediated transport and cholesterol metabolism and homeostasis. On the light of our results, the antileishmanial activity of VP343 is presumably associated with (1) a defect in the fusion of *Leishmania*-containing PVs with late endosome and lysosomal compartments, caused by the interference with membrane fusion regulators (such as Pik3c3) and/or microtubule-dependent vacuolar movements, by targeting tubulin deacetylases (such as Sirt2), and/or disrupting cholesterol homeostasis, resulting in a defective PV maturation. Furthermore, (2) VP343-induced ROS production within host cells, which would be toxic for intracellular *Leishmania*, and (3) epigenetic regulation, which may result from targeting of histone deacetylases (such as Sirt2), may also contribute to VP343 antileishmanial activity.

In future works, a more detailed investigation of the different host cell pathways targeted by VP343 determined in the current study would allow to identify the direct host molecular target(s) that bind to V343 within the host cell. Pharmacomodulations of the compound may also be considered in order to optimize its affinity for the identified target(s), based on the analysis of their molecular structure, as well as its antileishmanial activity.

### Limitations of the study

In this study, the mechanism of action of VP343 was investigated on RAW264.7 macrophages, in line with our previous work that described the anti-leishmanial activity of this compound on this same cell line.^19^ Moreover, the RAW264.7 cell line is a suitable host cell model widely used in the literature to study *Leishmania* infection.^10^^,^^13^^,^^15^^,^^38^ Although more physiologically relevant than macrophage cell lines, primary macrophages are usually associated with several drawbacks including ethical constraints, impossibility of cell storage, limited amount of cells, and heterogeneity among the isolated monocytes, with a percentage of adherent cells that will transform into functional macrophages, which could be as low as 1%,^57^ and among the differentiated macrophages.^58^ Conversely, cell lines, such as RAW264.7 macrophages, are much less concerned by ethical issues and have homogeneous background and a stable phenotype allowing high reproducibility of the experiments and thus constitute a reasonable alternative model to primary cells. Regardless of these differences, further analyses using primary cells, such as bone-marrow-derived macrophages, may be useful to confirm our results obtained using RAW264.7 and to avoid any biases associated with the host cell model.

## STAR★Methods

### Key resources table

**Table:** 

REAGENT or RESOURCE	SOURCE	IDENTIFIER
**Antibodies**		

Rabbit anti-EEA-1 polyclonal antibody	Thermo-Fisher Scientific	Cat# PA5-29013; RRID: AB_10694097
Goat anti-rabbit IgG, Alexa-594 conjugated	Thermo-Fisher Scientific	Cat# R37117; RRID: AB_10374440
Goat anti-Rat IgG, Alexa-594 conjugated	Thermo-Fisher Scientific	Cat# A-11007; RRID: AB_141374
Rat anti-Lamp-1 monoclonal antibody (1D4B)	DSHB	Registry ID: AB_528127; RRID: AB_10807835
Rabbit anti-Rab7 polyclonal antibody	Merck	Cat# R4779; RRID: AB_477460
Rabbit anti-Sec22b polyclonal antibody	Thermo-Fisher Scientific	Cat# OSS00040W; RRID: AB_2428132
Recombinant Anti-GAPDH antibody	Abcam	Cat# ab181602; RRID: AB_2630358
Goat anti-Rat IgG Secondary Antibody, HRP	Thermo-Fisher Scientific	Cat# A10549; RRID: AB_933896
Goat anti-Rabbit IgG Secondary Antibody, HRP	Thermo-Fisher Scientific	Cat# A16110; RRID: AB_1965959

**Chemicals, peptides, and recombinant proteins**		

N-(5-bromo-2-methoxybenzyl)-N-methyladamantan-1-amine (VP343)	Synthetized as previously described^19^	N/A
Dulbecco's Modified Eagle's Medium complete medium (DMEM)	Thermo-Fisher Scientific	Cat# 41965039
M199 medium	Merck	Cat# M5017
Penicillin-Streptomycin Solution	Merck	Cat# P0781
P3 primary cell buffer	Lonza Bioscience	Cat# V4XP-3032
Hygromycin	Merck	Cat# H3274

**Critical commercial assays**		

Cholesterol Assay Kit (Filipin III, U-18666A)	Abcam	Cat# ab133116
Amplex Red assay kit	Thermo-Fisher Scientific	Cat# A12216
Bradford protein assay kit	Thermo-Fisher Scientific	Cat# A55866
CellROX® Deep Red Oxidative Stress Reagents	Thermo-Fisher Scientific	Cat# C10422
Click-iT™ TUNEL Alexa Fluor™ 594 Imaging Assay	Thermo-Fisher Scientific	Cat# C10246
HiPerFect Transfection Reagent	Qiagen	Cat# 301704

**Experimental models: Cell lines**		

RAW 264.7 macrophage	ATCC	TIB-71
*Leishmania infantum*	National Center of Reference for Leishmaniasis (Montpellier)	(MHOM/FR/2008/LEM5700)

**Oligonucleotides**		

siRNAs targeting Scarb2, See Table S1	Qiagen	Cat# 1027416
siRNAs targeting Pik3c3, See Table S1	Qiagen	Cat# 1027416
siRNAs targeting Sirt2, See Table S1	Qiagen	Cat# 1027416
siRNAs targeting MvK, See Table S1	Qiagen	Cat# 1027416
AllStars Negative Control siRNA, See Table S1	Qiagen	Cat# SI03650318

**Recombinant DNA**		

pNUS-GFPcH plasmid	Provided by Dr. Emmanuel Tétaud^59^	N/A

**Software and algorithms**		

SP8 LAS X software, Version 3.6	Leica, Germany	https://www.leica-microsystems.com/fr/produits/logiciel-du-microscope/p/leica-las-x-ls/
ImageJ (Version 1.52q)	NIH	https://imagej.nih.gov/ij/index.html
Cytoscape open-source software platform (version 3.8.0)	Cytoscape	https://cytoscape.org/what_is_cytoscape.html
GraphPad prism 8	GraphPad by Dotmatics	https://www.graphpad.com/features
Perseus software	Max Planck Institute of Biochemistry	https://maxquant.net/perseus/

### Resource availability

#### Lead contact

Further information and requests for resources and reagents should be directed to and will be fulfilled by the lead contact, Sébastien POMEL (sebastien.pomel@universite-paris-saclay.fr).

#### Materials availability

This study did not generate new unique reagents.

### Experimental model and study participant details

#### Cell cultures

Promastigotes of *L. infantum* (MHOM/FR/2008/LEM5700) were grown in the dark at 27°C in M199 complete medium containing M199 medium supplemented with 100 μM adenosine, 0.5 mg/L hemin, 40 mM Hepes pH 7.4 and 10 % Heat Inactivated Foetal Bovine Serum (HIFBS). Differentiation of *L. infantum* promastigotes into axenic amastigotes was performed by incubating late log promastigotes in M199 complete medium pH 5.5, supplemented with 20 % HIFBS, at 37°C with 5 % CO_2_.^60^

The mouse monocyte/macrophage cell line RAW 264.7 was maintained at 37°C in a humidified atmosphere containing 5 % CO_2_, in Dulbecco's Modified Eagle's Medium complete medium (DMEM) supplemented with penicillin-streptomycin (100 U/mL) and 10 % HIFBS.

### Method details

#### Parasite transfection

In 100 μL of P3 primary cell buffer, 5 x 10^7^ log-phase parasites were mixed with 10 μg of pNUS-GFPcH plasmid,^59^ and transfected using the FP-100 program with the 4D-Nucleofector™ system (Lonza, Colmar, France). Parasites expressing GFP were further cloned twice in 1 % agar containing M199 complete medium with 50 μg/mL hygromycin. The selected parasites expressed invariably GFP for at least 10 passages in the absence of hygromycin. All experiments described below were performed in absence of hygromycin.

#### Treatment and infection of macrophages

For treatment and infection of RAW 264.7 macrophages, cells were seeded in 24 well plates, containing 12 mm round glass coverslips and incubated at 37°C under 5 % CO_2_ atmosphere. The next day, macrophages, reaching 80-85 % of confluence at that time, were treated by adding VP343 at 10 μM in DMEM complete medium. For infection of VP343-treated cells, RAW 264.7 macrophages were incubated with the compound for 1 h at 37°C, with 5 % CO_2_ prior to their infection. Unless specified in the text, the compound was maintained in the culture medium during all the course of infection. The VP343-treatment of cells before their infection was performed in order to ensure the presence of the compound during the early stages of infection, i.e. installation of the parasites within the PVs. Treated and untreated macrophages were infected by a co-incubation with *L. infantum* axenic amastigotes, at a parasite to macrophage ratio of 16:1, at 37°C with 5% CO_2_ for the incubation time specified in the text.

#### Immunofluorescence, cytochemical labeling and imaging

At the indicated time post-infection, coverslips were washed twice with pre-warmed PBS to remove non-internalized parasites. Cells were then fixed with 4% paraformaldehyde in PBS for 10 min at room temperature. After 3 washes with PBS, cell membranes were permeabilized by adding a solution of 0.1% Triton X-100 in PBS for 12 min at room temperature. Coverslips were then washed 3 times with PBS and blocked by adding 3 % Bovine Serum Albumin (BSA) in PBS for 1 h and then washed 3 times with 0.3 % BSA in PBS. Coverslips were incubated for 1 h at 37°C with one of the following primary antibodies diluted in 0.3 % BSA in PBS: anti-EEA-1 (1:500), anti-Rab7 (1:100) or anti-Lamp-1 (1:11).

Coverslips were then washed with 0.3 % BSA in PBS and incubated for 1 h at 37°C with the appropriate Alexa-594 conjugated secondary antibody (1:500 in 0.3 % BSA in PBS). For cholesterol, staining cells were incubated for 1 h at room temperature with Filipin III diluted 1:100 in cholesterol detection assay buffer. After staining, cells were washed three times in 0.3 % BSA in PBS. Coverslips were then incubated with 0.1 mg/mL Hoechst in BPS, for 15 min at room temperature, to stain the nuclei. Coverslips were washed twice with PBS, once with ultrapure water and then mounted on glass slides in Mowiol mounting medium.

Images were mainly performed using Leica TCS SP8 inverted confocal microscope (Leica, Germany), except for Filipin imaging that were done using AXIO-OBSERVER Z1-COLIBRI videomicroscope (Carl Zeiss, Germany), with a HC Plan-Apochromat 63x/1.4 NA oil immersion objective lens. The confocal microscope was equipped with a 405 nm diode for DAPI (nuclei) excitation and a WLL Laser (594 nm excitation for Alexa-594). Blue and red fluorescence emission were collected respectively with a 410-460 and a 600-760 nm wide emission slits using a sequential mode. The pinhole was set at 1.0 Airy unit giving an optical slice thickness of 0.89μm.

Image analysis was performed using SP8 LAS X software and ImageJ software (version 1.52q, NIH). Colocalization was estimated using Pearson’s correlation coefficient^61^ that was calculated by the JACoP plugin in ImageJ after background subtraction.

#### Determination of intracellular cholesterol content

Total intracellular cholesterol content was determined from cell lysates using the Amplex Red assay kit^62^ according to manufacturer's recommendations. Briefly, RAW 264.7 macrophages cultures were incubated for 5 h with serum-free culture medium containing or not 10 μM of VP343. Cells were then washed twice with PBS and centrifuged at 1000 *g* for 10 min at room temperature. Pellets were then resuspended in a cold lysis buffer containing 100 mM potassium phosphate, 50 mM NaCl, 5 mM cholic acid and 0.5 % Triton X-100, pH 7.4, and further sonicated with a sonicator (Vibra cell 72434, Bioblock scientific, Illkrich, France) at high-intensity with three cycles of 10 s using a microtip probe. The protein concentration of the cell lysate samples was measured by the Bradford method. Cell lysates were then incubated with Amplex-Red reagent for 1 h at 37°C, and fluorescence was measured using a SPARK multimode microplate reader (TECAN, Grödig, Austria) with excitation and emission wavelengths at 535-25 nm and 595-35 nm, respectively. The cholesterol values were normalized to total cellular protein levels. All results were then normalized to the mean cholesterol content of untreated sample.

#### Proteomic analysis

For proteomic analysis, Mass Spectrometry (MS) was performed on whole cell prepared from RAW 264.7 macrophages treated or not with 10 μM VP343 during 5 h or 16 h. For preparation of cell lysates, untreated and VP343-treated cells were scraped from culture plate, counted and then centrifuged. Cell pellets were resuspended in 1X Laemmli sample buffer (62.5 mM Tris, pH 6.8, 2 % SDS, 10 % glycerol, 5 % 2-mercaptoethanol) and boiled for 10 min at 100°C. For each sample, an amount equivalent to 2x10^5^ cells was deposited and separated to a short distance (∼50-100 mm) in a 10 % SDS-polyacrylamide gel. The protein bands were visualized with Coomassie Blue G-250 staining solution and subjected to trypsin digestion before MS analysis, as previously described.^63^ Trypsin-generated peptides were analyzed by nanoLC-MS/MS (liquid chromatography tandem mass spectrometry) using a nanoElute liquid chromatography system (Bruker) coupled to a timsTOF Pro mass spectrometer (Bruker). Peptides were loaded with solvent A on a trap column (nanoEase C18, 100 Å, 5 μm, 180 m × 20 mm) and separated on an Aurora analytical column (ION OPTIK, 25 cm × 75 μm, C18, 1.6 μm) with a gradient of 0–35% of solvent B for 100 min. Solvent A was 0.1% formic acid and 2% acetonitrile in water, and solvent B was 0.1% formic acid and 99.9% acetonitrile. MS and MS/MS spectra were recorded from m/z 100 to 1700 with a mobility scan range from 0.6 to 1.4 V s/cm^2^. MS/MS spectra were acquired with the PASEF (Parallel Accumulation Serial Fragmentation) ion mobility-based acquisition mode using a number of PASEF MS/MS scans set as 10. MS and MSMS raw data were processed and converted into mgf files with Data Analysis software (Bruker). Protein identifications were performed against SwissProt database and *Mus musculus* taxonomy using the MASCOT search engine (Matrix science, London, United Kingdom). Database searches were performed using the following parameters: specific trypsin digestion with two possible miscleavages; carbamidomethylation of cysteines as fixed modification and oxidation of methionines as variable modification. Peptide and fragment tolerances were 10 ppm and 0.05 Da, respectively. Protein identifications were validated when identified with at least two unique peptides in at least one replicate, identified with a score higher than the identity threshold, and a false-positive discovery rate of less than 1% (Mascot decoy option). Mass spectrometry based-quantification was performed by label-free quantification using MS1 ion intensities named XIC (for extracted ion current). MS raw files were analyzed with Maxquant software using the maxLFQ algorithm with default settings and 4D feature alignment corresponding to a match between run function including collisional cross sections (CCS) alignment. Normalization was set as default. All statistical analyses for MS were done on biological quadruplicates using Welch’s t-test with Perseus software (Max Planck Institute of Biochemistry) and proteins were filtered on a P-value <0.05 and a fold change larger than two.

The list of differentially expressed proteins was applied into STRING (version 11.5) database to generate a protein-protein network model on the basis of evidence sources (i.e. experiments, databases, text mining, co-expression, neighborhood, gene fusion and co-occurrence) and a minimal confidence score of 0.400. The constructed networks were then exported as text files and handled by using Cytoscape open-source software platform (version 3.8.0). The combined score for each interaction was represented by the width of the edge between two nodes.

#### RNA interference

Transfection of RAW 264.7 macrophages with siRNA was performed using the HiPerFect Transfection Reagent according to the manufacturer’s protocols. Briefly, cells seeded in 24-well plate containing sterile coverslips, at a density of 1x10^5^ cells/well, were transfected with siRNAs targeting Scarb2 gene, Mvk gene, Pik3c3 gene, Sirt2 gene or with negative control siRNA (Table S6**)**, and incubated for 24 h, at 37°C with 5% CO_2_, before infection with *L. infantum*, as described above. For each gene, we used a mix of 4 preselected siRNAs, each at 25 nM. For the AllStars negative control siRNA, we used one siRNA at 40 nM. At 24 h and 48 h post-infection, cells were processed for immunolabelling and imaging analysis, as mentioned above, to determine the parasite load and the level of Lamp1 recruitment to PVs.

#### Measurement of intracellular ROS

The measurement of Reactive Oxygen Species (ROS) in *L. infantum* infected or uninfected RAW 264.7 macrophages, treated or not with VP343 (according to the STAR Methods detailed above), was done using the CellROX® Deep Red Oxidative Stress Reagent. This cell permeable reagent exhibits strong fluorogenic signal once reduced or upon oxidation. At 5 h and 16 h post-infection, cells were incubated with 5 μM CellROX® Deep Red Reagent for 30 min at 37°C with 5% CO_2_. Cells were then washed three times with PBS. The fluorescence intensity was measured using a SPARK multimode microplate reader (TECAN, Grödig, Austria) with excitation and emission wavelengths at 620-20 nm and 680-30nm, respectively.

#### *In situ* measurement of DNA fragmentation by TUNEL

To assess intracellular *Leishmania* apoptosis, we applied the Click-iT™ TUNEL Alexa Fluor® Assay to detect DNA fragmentation in apoptotic parasites. Untreated and VP343-treated RAW 264.7 macrophages, previously infected by *L. infantum*, were fixed with 4 % paraformaldehyde and permeabilized with 0.25 % Triton X-100 for 20 min at room temperature. All enzymes and buffers were further added according to the manufacturer’s instructions. Briefly, cells were incubated for 60 min at 37°C with TdT reaction cocktail composed of TdT reaction buffer, the enzyme terminal deoxynucleotidyl transferase (TdT) and EdUTP nucleotide mixture. After two washes in 3 % BSA in PBS, cells were incubated with Click-iT™ reaction cocktail (Click-iT™ reaction buffer with Click-iT™ reaction buffer additive) for 30 min at room temperature and protected from light. Cell nuclei were then labelled with 0.1 μg/mL Hoechst in PBS. Parasite DNA fragmentation was detected by cell imaging using Leica TCS SP8 inverted confocal microscope (Leica, Germany) with excitation/emission wavelengths at 590/617 nm. Parasites were considered as apoptotic when their nuclear and/or kinetoplast DNA were labelled with TUNEL.

### Quantification and statistical analysis

All data were analyzed by the Kolmogorov-Smirnov normality test. Data with normal distribution were analysed using Student’s *t*-test. Data whose distribution were not considered normal were analyzed using the non-parametric Mann-Whitney's test. Data were considered significantly different at *p* values ≤ 0.05 (≤ 0.05 ∗, ≤ 0.01 ∗∗, ≤ 0.001 ∗∗∗). Data analysis and the generation of graphs were performed using GraphPad prism 8 software (San Diego, CA, USA).

## Data Availability

• This paper does not report original code. • All data reported in this paper will be shared by the lead contact upon request. • Any additional information required to reanalyze the data reported in this paper is available from the lead contact upon request.
