# Supplementary material for: Deciphering the mechanism of action of VP343, an antileishmanial drug candidate, in Leishmania infantum
Source: iScience. 2023 Oct 5;26(11):108144. doi: 10.1016/j.isci.2023.108144 (PMC10616420; doi:10.1016/j.isci.2023.108144)
Supplement: Document S1. Figures S1–S3 and Tables S1–S6 [file mmc1.pdf]

iScience, Volume *26*

Supplemental information

Deciphering the mechanism of action of VP343, an antileishmanial drug candidate, in *Leishmania infantum*

Sameh Obeid, Eloisa Berbel-Manaia, Valérie Nicolas, Indira Dennemont, Julien Barbier, Jean-Christophe Cintrat, Daniel Gillet, Philippe M. Loiseau, and Sébastien Pomel

Supplemental Information

Figure S1 – The effect of VP343 compound on the growth, the differentiation and the virulence of *L. infantum*, Related to Figure 1

(A) Growth curve of *L. infantum* promastigotes in the presence of 10 µM of VP343. The arrow in the graph corresponds to the moment when the parasites were taken to be differentiated in axenic amastigotes. (B) Proportion of *L. infantum* amastigotes after differentiation in the presence or absence of VP343. This proportion was of ≈80% in both cases. Data are means ± SD of triplicate measurements. (C) RAW264.7 macrophages were infected with differentiated untreated *L. infantum* parasites (condition 1), or with parasites that were pre-incubated during 1h with 10 µM VP343 (condition 2 & 3). In conditions 1 & 2 the infection occurred in the absence of VP343, while in condition 3 the VP343 was kept in the culture medium during all the infection time. At 24h post-infection, cells were processed for imaging in order to determine the infection rate for each condition (i.e. ratio parasite/cell). The figure represents one out of the three independent experiments that were done in duplicate. "n" represents the number of analyzed cells. Statistical analysis was done using the Student's t-test. *p < 0.05; **p<0.01.

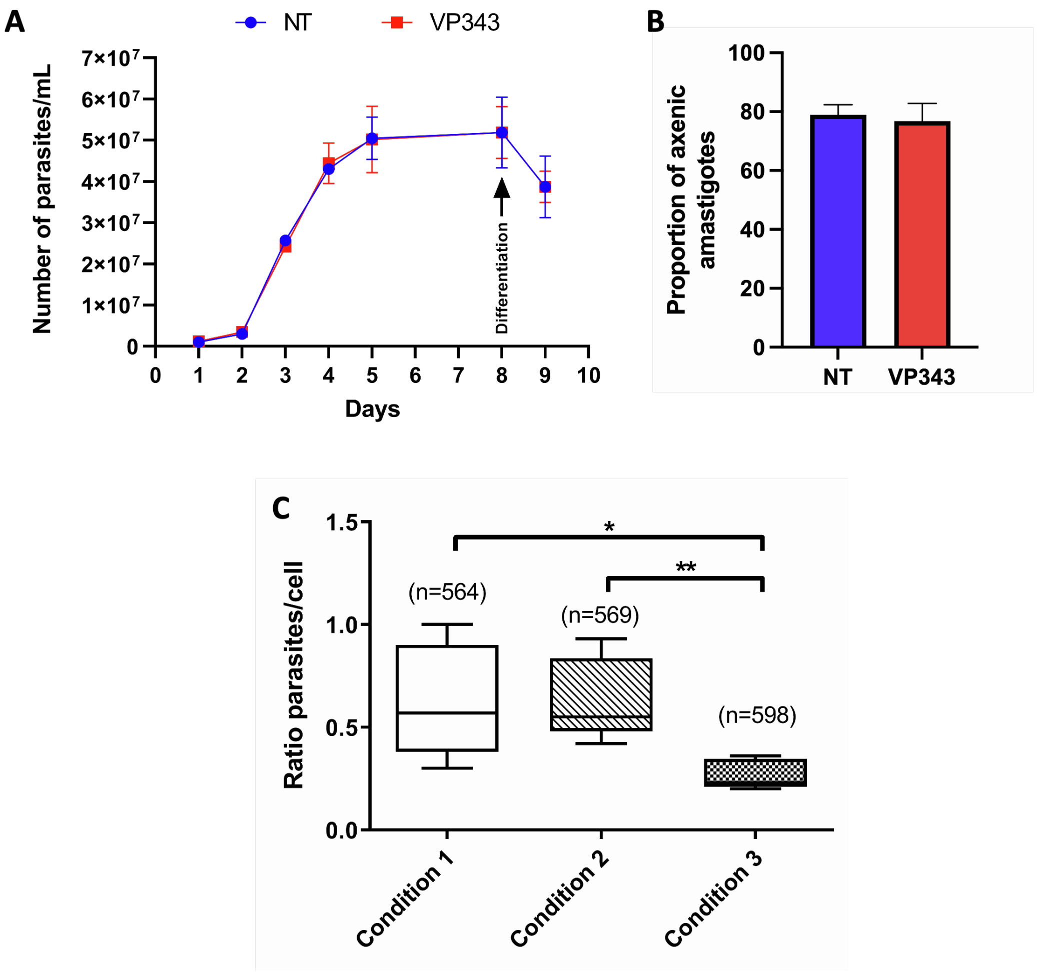

Figure S2 – Western-Blot analysis of the host-cell vesicular trafficking markers, Related to Figure 2

*Protein extracts were prepared from macrophages treated or not with 10 µM VP343 for 5h or 16h. Briefly, cells were scraped from culture plate, centrifuged and pellets were resuspended in 1X Laemmli sample buffer containing 2% SDS, 10% glycerol, 5% 2-mercaptoethanol, 0.01% bromophenol blue and 0.063 M Tris HCl, pH 6.8, and boiled for 10 min at 100°C. The cell extracts were analyzed by Western blot. Specific antibodies were used for the detection of each protein: anti-EEA-1(d 1:3000; Thermo-Fisher Scientific, Illkrich, France), anti-Lamp-1 (1D4B; d 1:100; DSHB, Iowa city, IA, USA), anti-Sec22b (d 1:1000; Thermo-Fisher Scientific, Illkrich, France), anti-Rab7 (d 1:2000; Sigma-Aldrich, Saint Quentin Fallavier, France) and anti-GAPDH (d 1:5000; Abcam, Amsterdam, Netherlands). For the detection, HRP-conjugated anti-rat (d 1: 3000) or anti-rabbit (d 1:10 000) secondary antibodies (Thermo-Fisher Scientific, Illkrich, France) were used. (A) Representative Western blots of the different proteins analyzed. (B) The relative levels of each protein were determined by normalizing band intensities, measured using ImageJ software (*version 1.52q, NIH*), with those of GAPDH (control). The experiment was done in quadruplicate, except for Sec22b where analysis was done in triplicate.* Data are means ± SD. *The results showed no significant differences in the expression level of EEA-1, Lamp-1, Sec22b and Rab7 between untreated and VP343-treated cells. NT: Non Treated cells; T: VP343-treated cells*

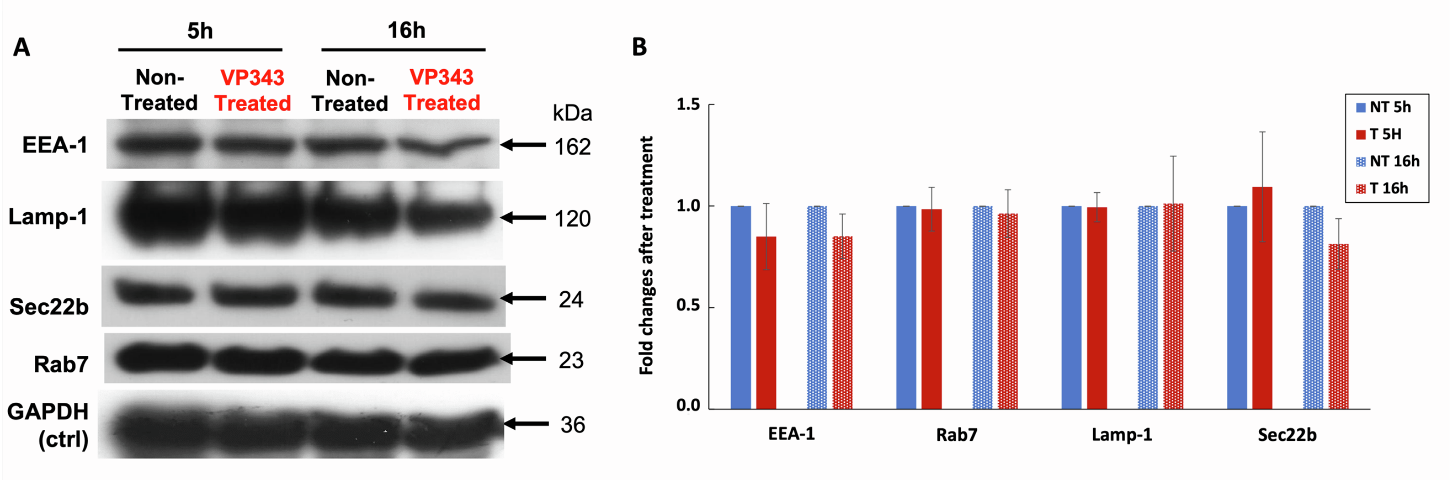

Figure S3 – Effect of VP343 on the cholesterol accumulation around intracellular *Leishmania infantum*, Related to Figures 3 and 4

Macrophages were infected with *L. infantum* and further treated or not with 10 µM of VP343 for 5 h. Cells were then fixed and incubated with Filipin III and Hoechst. (A) The white arrows in the fluorescence images indicate intracellular *L. infantum* amastigotes. The uptake and accumulation of free cholesterol around intracellular *Leishmania* was observed as a halo. Scale bar= 10 µm. (B) The analysis of the fluorescence signal intensities of the halo in untreated and VP343-treated cells was done in duplicate for a total 30 intracellular amastigotes. Data are means ± SD. No significant difference was observed between untreated and VP343-treated cells. Statistical analysis was done using the Student's t-test.

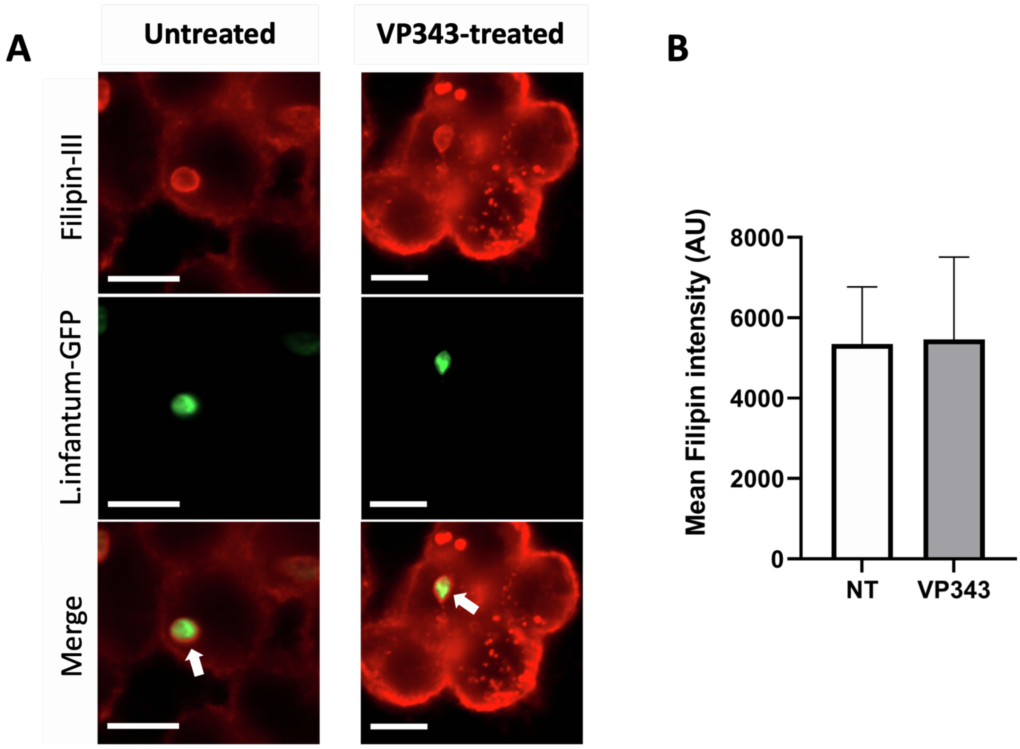

Table S1: Cytotoxicity of VP343 against RAW264.7 macrophages, Related to Figure 1

The CC_{50}, corresponding to the concentration of VP343 inhibiting RAW264.7 macrophage metabolic activity by 50%, was determined after 24 h, 48 h or 72 h of incubation.

Cytotoxicity	Incubation time		
	24 h	48 h	72 h
CC_{50} ± SD (µM)	165.5 ± 2.9	81.8 ± 10.4	45.6 ± 6.5

Table S2 – List of proteins identified by Mass-spectrometry analysis downregulated at 5 h post-treatment of RAW264.7 with VP343, Related to Figure 5

Protein IDs	Protein names	Gene names	Sequence coverage [%]	Mol. weight [kDa]	Fold decrease after treatment
P41105	60S ribosomal protein L28	Rpl28	16.1	15.733	17.5
Q3USZ8	Deleted in autism protein 1 homolog	Dipk2a	22.6	49.462	8.3
P70362	Ubiquitin fusion degradation protein 1 homolog	Ufd1l	12.1	34.481	7.3
Q8R4R6	Nucleoporin NUP53	Nup35	35.1	34.785	7.2
Q8VDQ8	NAD-dependent protein deacetylase sirtuin-2	Sirt2	20.3	43.256	6.2
Q3ULW8		Parp3	6.2	59.948	5.6
P28076	Proteasome subunit beta type-9	Psmb9	21.9	23.396	5.0
Q80VI1	E3 ubiquitin-protein ligase TRIM56	Trim56	9.9	79.512	4.9
Q8CH72	E3 ubiquitin-protein ligase TRIM32	Trim32	8.4	72.057	4.9
Q8K4L0	ATP-dependent RNA helicase DDX54	Ddx54	9.5	97.747	4.5
Q9DCF9	Translocon-associated protein subunit gamma	Ssr3	7.6	21.064	4.3
Q9QZM0	Ubiquilin-2	Ubqln2	13	67.35	4.3
O35114	Lysosome membrane protein 2	Scarb2	14.2	54.043	4.2
Q9R008	Mevalonate kinase	Mvk	15.4	41.877	4.0
Q3UQN2	F-BAR domain only protein 2	Fcho2	14.8	88.733	3.9
Q9DC48	Pre-mRNA-processing factor 17	Cdc40	12.1	65.46	3.8
P51125	Calpastatin	Cast	13.5	84.921	3.7
Q9DB25	Dolichyl-phosphate beta-glucosyltransferase	Alg5	13.6	36.79	3.6
Q8VEH6	COBW domain-containing protein 1	Cbwd1	22.4	43.771	3.5
Q8R2U4	N-terminal Xaa-Pro-Lys N-methyltransferase 1;N-terminal Xaa-Pro-Lys N-methyltransferase 1, N-terminally processed	Ntmt1	9.4	25.42	3.5
Q9D125	28S ribosomal protein S25, mitochondrial	Mrps25	13.5	19.92	3.5
Q60866	Phosphotriesterase-related protein	Pter	18.9	39.218	3.4
P51670	C-C motif chemokine 9;CCL9(29-101);CCL9(30-101);CCL9(31-101)	Ccl9	17.2	13.871	3.3
Q9D0C4	tRNA (guanine(37)-N1)-methyltransferase	Trmt5	9.2	56.794	3.2
P62331	ADP-ribosylation factor 6	Arf6	44.6	20.082	3.1
P54729	NEDD8 ultimate buster 1	Nub1	16.1	70.306	3.1
P56477	Interferon regulatory factor 5	Irf5	39.4	56.004	3.0

P24860	G2/mitotic-specific cyclin-B1	Ccnb1	10.7	48.051	3.0
O70591	Prefoldin subunit 2	Pfdn2	18.8	16.534	3.0
Q8K0G5	Protein TSSC1	Tssc1	22.3	43.126	2.9
Q8R5L3	Vam6/Vps39-like protein	Vps39	8.7	101.69	2.8
Q8BFP9	[Pyruvate dehydrogenase (acetyl-transferring)] kinase isozyme 1, mitochondrial	Pdk1	18.0	48.994	2.8
Q6PF93	Phosphatidylinositol 3-kinase catalytic subunit type 3	Pik3c3	7.6	101.49	2.7
A6H630	Protein-glutamate O-methyltransferase	Armt1	3.9	50.548	2.6
Q66GT5	Phosphatidylglycerophosphatase and protein-tyrosine phosphatase 1	Ptpmt1	40.9	21.942	2.6
O70566	Protein diaphanous homolog 2	Diaph2	4.0	124.87	2.6
P97930	Thymidylate kinase	Dtymk	22.6	23.914	2.6
Q62465	Synaptic vesicle membrane protein VAT-1 homolog	Vat1	20.7	43.096	2.6
Q9QZN4	F-box only protein 6	Fbxo6	8.5	34.492	2.6
Q9DC70	NADH dehydrogenase [ubiquinone] iron-sulfur protein 7, mitochondrial	Ndufs7	28.1	24.683	2.6
A6H8H2	DENN domain-containing protein 4C	Dennd4c	8.9	211.46	2.4
Q8BHD7	Polypyrimidine tract-binding protein 3	Ptbp3	44.9	56.7	2.4
Q8K301	Probable ATP-dependent RNA helicase DDX52	Ddx52	21.7	67.473	2.4
Q9CQT2	RNA-binding protein 7	Rbm7	19.6	30.148	2.4
P53702	Cytochrome c-type heme lyase	Hccs	25.4	30.977	2.4
Q62384	Zinc finger protein ZPR1	Zpr1	15.0	50.714	2.3
Q8BFR4	N-acetylglucosamine-6-sulfatase	Gns	26.3	61.174	2.3
Q5HZI9	Solute carrier family 25 member 51	Slc25a51	6.4	33.689	2.2
Q9CQV1	Mitochondrial import inner membrane translocase subunit TIM16	Pam16	21.6	13.784	2.2
Q6P1H6	Ankyrin repeat and LEM domain-containing protein 2	Ankle2	4.4	106.2	2.2

Table S3 – List of proteins identified by Mass-spectrometry analysis upregulated at 5 h post-treatment of RAW264.7 with VP343, Related to Figure 5

Protein IDs	Protein names	Gene names	Sequence coverage [%]	Mol. weight [kDa]	Fold increase after treatment
O88455	7-dehydrocholesterol reductase	Dhcr7	4.7	53.918	4.3
Q5XJY4	Presenilins-associated rhomboid-like protein, mitochondrial;P-beta	Parl	5.3	41.963	4.0
Q5SUC9	Protein SCO1 homolog, mitochondrial	Sco1	10.2	31.617	3.9
Q8BG67	Protein EFR3 homolog A	Efr3a	5	92.612	3.4
Q3U0M1	Trafficking protein particle complex subunit 9	Trappc9	2.4	128.23	3.0
Q9Z2V5	Histone deacetylase 6	Hdac6	1.2	125.79	3.0
P83093	Stromal interaction molecule 2	Stim2	2.5	83.924	2.9
Q8BHJ5	F-box-like/WD repeat-containing protein TBL1XR1	Tbl1xr1	25.1	55.66	2.9
P35951	Low-density lipoprotein receptor	Ldlr	13.7	94.946	2.8
Q9Z1K5	E3 ubiquitin-protein ligase ARIH1	Arih1	7	64.016	2.8
P26450	Phosphatidylinositol 3-kinase regulatory subunit alpha	Pik3r1	13.3	83.516	2.7
Q91YE7	RNA-binding protein 5	Rbm5	3.1	92.31	2.7
P31001	Desmin	Des	10.2	53.497	2.5
P05977;P09542	Myosin light chain 1/3, skeletal muscle isoform;Myosin light chain 3	Myl1;Myl3	8.5	20.594	2.5
Q5PSV9	Mediator of DNA damage checkpoint protein 1	Mdc1	2.9	184.67	2.4
O70201	Baculoviral IAP repeat-containing protein 5	Birc5	10	16.297	2.3
Q03141	MAP/microtubule affinity-regulating kinase 3	Mark3	2.3	84.389	2.3
Q9CZ91	Serum response factor-binding protein 1	Srfbp1	7	48.745	2.2
Q925J9	Mediator of RNA polymerase II transcription subunit 1	Med1	1.8	167.14	2.2
Q05443; P51885	Lumican	Lum	10.2	38.756	2.1
Q9R1X4	Protein timeless homolog	Timeless	7.7	137.5	2.1
Q8R3Q6	Coiled-coil domain-containing protein 58	Ccdc58	11.8	16.665	2.0

Table S4 – List of proteins identified by Mass-spectrometry analysis downregulated at 16 h post-treatment of RAW264.7 with VP343, Related to Figure 5

Protein IDs	Protein names	Gene names	Sequence coverage [%]	Mol. weight [kDa]	Fold decrease after treatment
P14234	Tyrosine-protein kinase Fgr	Fgr	21.5	58.866	2.0
Q9QX60	Deoxyguanosine kinase, mitochondrial	Dguok	9.4	32.281	2.0
Q62018	RNA polymerase-associated protein CTR9 homolog	Ctr9	10.5	133.41	2.0
Q7TSI1	Pleckstrin homology domain-containing family M member 1	Plekhm1	5.5	118.53	2.0
Q91WT8; P86049; Q5YD48	RNA-binding protein 47	Rbm47	11.9	64.061	2.3
Q3TCN2	Putative phospholipase B-like 2;Putative phospholipase B-like 2 28 kDa form;Putative phospholipase B-like 2 40 kDa form;Putative phospholipase B-like 2 15 kDa form	Plbd2	23.2	66.289	2.3
Q921C5;Q8BR07	Protein bicaudal D homolog 2	Bicd2	10.7	93.39	2.4
Q6NVF4	DNA helicase B	Helb	3.7	121.47	2.4
Q6P2L6	Histone-lysine N-methyltransferase NSD3	Whsc1l1	4.9	161	2.4
Q8C3R1	BRCA1-associated ATM activator 1	Brat1	6.3	89.085	2.5
Q99N84	28S ribosomal protein S18b, mitochondrial	Mrps18b	14.6	28.702	3.0
P60898	DNA-directed RNA polymerase II subunit RPB9	Polr2i	28.8	14.523	3.2
Q149F1	RNA pseudouridylate synthase domain-containing protein 2	Rpusd2	5.1	61.534	3.3
Q8VI84	Nucleolar complex protein 3 homolog	Noc3l	10.2	93.21	3.3
Q9D0Q7	39S ribosomal protein L45, mitochondrial	Mrpl45	17.6	35.41	3.6
A2BH40	AT-rich interactive domain-containing protein 1A	Arid1a	1.4	242.09	3.8
Q80WC7	Arf-GAP domain and FG repeat-containing protein 2	Agfg2	15.2	48.967	4.0
Q922Q1	Mitochondrial amidoxime reducing component 2	44257	26.6	38.194	6.2
Q810A3	Tetratricopeptide repeat protein 9C	Ttc9c	24	19.997	7.0

Table S5 – List of proteins identified by Mass-spectrometry analysis upregulated at 16 h post-treatment of RAW264.7 with VP343, Related to Figure 5

Protein IDs	Protein names	Gene names	Sequence coverage [%]	Mol. weight [kDa]	Fold increase after treatment
O55029	Coatomer subunit beta	Copb2	22.3	102.45	2.1
Q9QY81	Nuclear pore membrane glycoprotein 210	Nup210	2.9	204.1	2.1
P58064	28S ribosomal protein S6, mitochondrial	Mrps6	28	14.308	2.3
P60762	Mortality factor 4-like protein 1	Morf4l1	34.3	41.492	2.4
P35951	Low-density lipoprotein receptor	Ldlr	13.7	94.946	2.6
A6H611	Mitochondrial intermediate peptidase	Mipep	8.6	80.851	2.7
Q6P9R1	ATP-dependent RNA helicase DDX51	Ddx51	7	70.367	3.1
Q9D666	SUN domain-containing protein 1	Sun1	5.7	101.98	3.2
Q8BHE8	Uncharacterized protein C2orf47 homolog, mitochondrial		12	32.985	3.3
P97930	Thymidylate kinase	Dtymk	22.6	23.914	3.7
Q9CRD2	ER membrane protein complex subunit 2	Emc2	32	34.934	3.7
Q8C3Q9	Caspase-9;Caspase-9 subunit p35;Caspase-9 subunit p10	Casp9	11.7	49.979	3.8
B2RUP2	Protein unc-13 homolog D	Unc13d	4.1	123.12	4.1
Q3TYA6	M-phase phosphoprotein 8	Mphosph8	3	97.466	4.1
Q9Z2Q5	39S ribosomal protein L40, mitochondrial	Mrpl40	20.9	24.301	4.5
Q9D6U8	Protein FAM162A	Fam162a	8.4	17.725	4.8
Q80SU7	Interferon-induced very large GTPase 1	Gvin1	1.3	280.81	5.3
O55234	Proteasome subunit beta type-5	Psmb5	14.8	28.532	6.4
P21855	B-cell differentiation antigen CD72	Cd72	25.7	40.347	6.6
Q8BSS9;P60469	Liprin-alpha-2;Liprin-alpha-3	Ppfia2;Ppfia3	2.3	143.23	7.4
Q61103	Zinc finger protein ubi-d4	Dpf2	13.6	44.229	7.9

Table S6 – List of the RNA sequences targeted by the siRNAs used in the RNA interference experiments, Related to Figure 6

Targets	siRNA	Forward
**Scarb2**	Mm_Scarb2_8 FlexiTube siRNA SI02731876	5’-CGAGAAGAAUAUGGUAUUA-3’
	Mm_Scarb2_7 FlexiTube siRNA SI02707831	5’-GGCAAUUCUGCUUUAUAAA-3’
	Mm_Scarb2_6 FlexiTube siRNA SI02686124	5’-CGACUUUGGUUGUCACCAA-3’
	Mm_Scarb2_5 FlexiTube siRNA SI02666391	5’-GGAAGUAUUUAAUAAACUU-3’
**Pik3c3**	Mm_Pik3c3_4 FlexiTube siRNA SI01378468	5’-GAGGAUUAUGUAUAUGAAA-3’
	Mm_Pik3c3_3 FlexiTube siRNA SI01378461	5’-GCUGUGACCUGGACAUCAA-3’
	Mm_Pik3c3_2 FlexiTube siRNA SI01378454	5’-GCUCCCAAAUUAUAACCAA-3’
	Mm_Pik3c3_1 FlexiTube siRNA SI01378447	5’-GAAAGAUAGUCAAACUUCA-3’
**Mvk**	Mm_Mvk_4 FlexiTube siRNA SI01320788	5’-GGUCAUCCUCCAUGGAGAA-3’
	Mm_Mvk_3 FlexiTube siRNA SI01320781	5’-GGAACAGUACCUCGUACUA-3’
	Mm_Mvk_2 FlexiTube siRNA SI01320774	5’-GGACGAUGUCUUCCUUGAA-3’
	Mm_Mvk_1 FlexiTube siRNA SI01320767	5’-GAGGAAGAUCUGAAGUCAA-3’
**Sirt2**	Mm_Sirt2_7 FlexiTube siRNA SI04957050	5’-CACGGCUGCUCAUUAACAA-3’
	Mm_Sirt2_5 FlexiTube siRNA SI02718037	5’-GAGGGAGCAUGCCAACAUA-3’
	Mm_Sirt2_2 FlexiTube siRNA SI00220122	5’- GCUAAAUCAAAUUAACCUA-3’
	Mm_Sirt2_1 FlexiTube siRNA SI00220115	5’- AGAAUAAGGCAUUUCUCUA-3’
**AllStars Negative Control siRNA**	AllStars Negative Control siRNA SI03650318	5’-GGGUAUCGACGAUUACAAA-3’
